# Insights into the Roles of Lipoteichoic Acids and MprF in Bacillus subtilis

**DOI:** 10.1128/mbio.02667-22

**Published:** 2023-02-06

**Authors:** Aurélie Guyet, Amirah Alofi, Richard A. Daniel

**Affiliations:** a Centre for Bacterial Cell Biology, Biosciences Institute, Faculty of Medical Sciences, Newcastle University, Newcastle upon Tyne, United Kingdom; Institut Pasteur

**Keywords:** LytE, MprF, PBP1, daptomycin, *Bacillus subtilis*, lipoteichoic acid

## Abstract

Gram-positive bacterial cells are protected from the environment by a cell envelope that is comprised of a thick layer of peptidoglycan that maintains cell shape and teichoic acid polymers whose biological function remains unclear. In Bacillus subtilis, the loss of all class A penicillin-binding proteins (aPBPs), which function in peptidoglycan synthesis, is conditionally lethal. Here, we show that this lethality is associated with an alteration of lipoteichoic acids (LTAs) and the accumulation of the major autolysin LytE in the cell wall. Our analysis provides further evidence that the length and abundance of LTAs act to regulate the cellular level and activity of autolytic enzymes, specifically LytE. Importantly, we identify a novel function for the aminoacyl-phosphatidylglycerol synthase MprF in the modulation of LTA biosynthesis in both B. subtilis and Staphylococcus aureus. This finding has implications for our understanding of antimicrobial resistance (particularly to daptomycin) in clinically relevant bacteria and the involvement of MprF in the virulence of pathogens such as methicillin-resistant S. aureus (MRSA).

## INTRODUCTION

**FIG 1:**
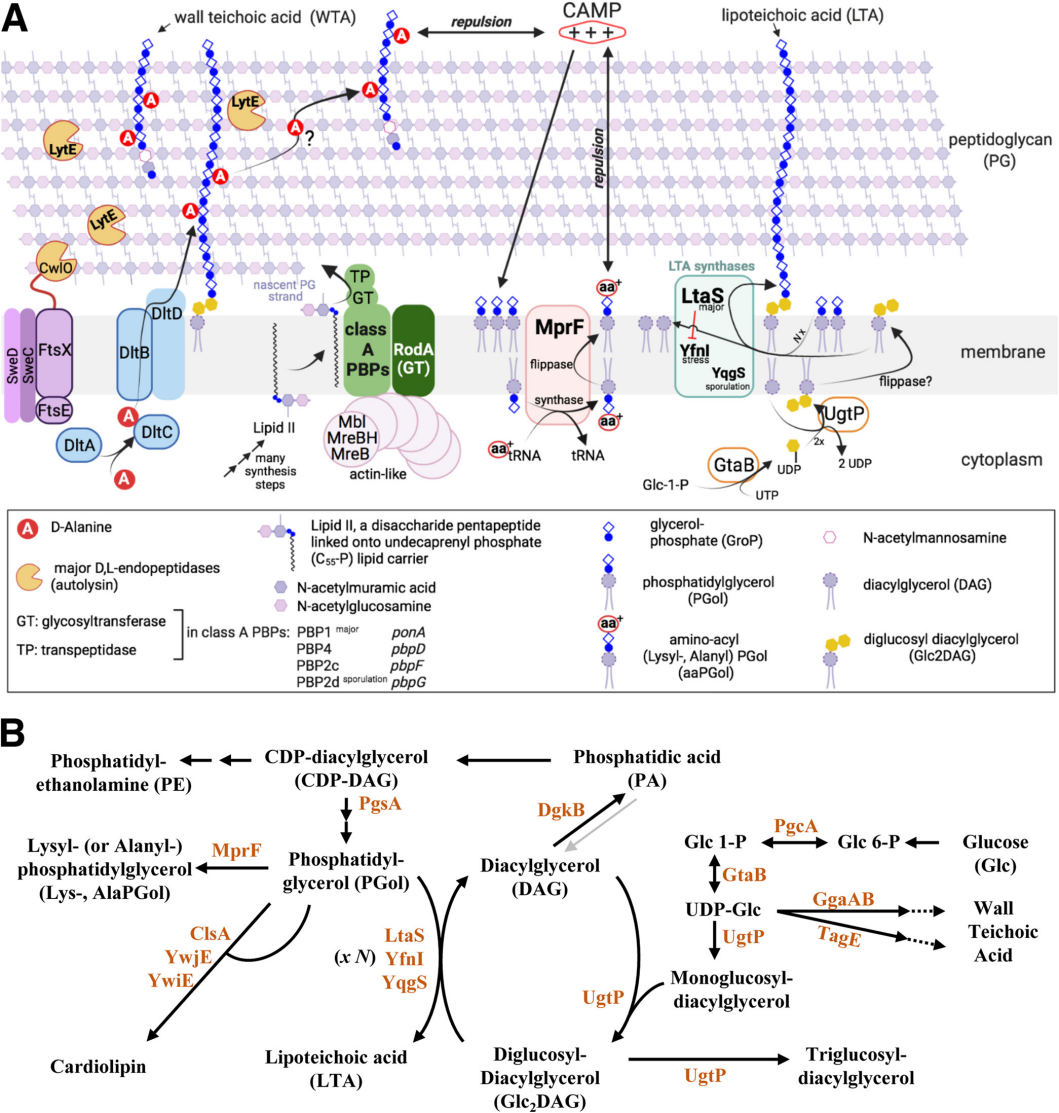
Representations of the cell envelope of the Gram-positive rod-shaped bacterium Bacillus subtilis and biosynthetic links between lipoteichoic acids and phospholipids. (A) Depiction of some of the cell envelope components and their functions in B. subtilis. This figure indicates the complexity of cell envelope synthesis due to the presence of enzymes that seemingly assume the same function. This redundancy of function has been identified in the reaction steps leading to the synthesis of the peptidoglycan (glycosyltransferases, 4 class A PBPs, and the essential RodA), the lipoteichoic acids (3 LTA synthases, where LtaS is the major enzyme), and the actin-like filaments that coordinate cell shape (3 Mre homologs, MreB/MreBH and Mbl, where the latter is essential in B. subtilis 168CA). The major autolysins CwlO and LytE (the absence of both is lethal to B. subtilis) cleave the peptidoglycan pentapeptides. The presence of aaPGols produced by MprF and the d-alanylation of teichoic acids by Dlt proteins are thought to create an electrostatic environment that protects the bacteria from CAMPs (cationic antimicrobial peptides). (Illustration created with BioRender.com.) (B) Schematic representation of the biosynthetic links among lipoteichoic acids, phospholipids, and glucolipids in B. subtilis (3, 83). LTAs consist of glycerolphosphate units anchored to the membrane by a glycolipid usually synthesized by UgtP. When grown in LB medium at 37°C, the majority of B. subtilis phospholipids are phosphatidylglycerol (37.5% ± 6.2%), phosphatidylethanolamine (30.5% ± 3.8%), Glc_2_DAG (9.6% ± 4.7%), and lysyl-phosphatidylglycerol (LysPGol) (7.8% ± 3.7%) produced by MprF (83). Recently, l- and *N*-succinyl-LysPGol and l- and d-AlaPGol were identified in B. subtilis (25). The gray arrow indicates a reaction step that might be bidirectional.

Gram-positive bacteria have a common cell envelope architecture that contributes to maintaining cell shape and protects the cell from environmental changes. The best-characterized component of the cell envelope is the peptidoglycan (PG), which forms a mesh-like structure enclosing the cell membrane (Fig. 1A). On this structure, anionic polymers are either covalently attached to PG *N*-acetylmuramic acids, known as wall teichoic acids (WTAs), or tethered to the external face of the membrane, named lipoteichoic acids (LTAs) (1–4). It is unclear whether LTAs are localized in a periplasm-like space between the membrane and PG or extend through the PG layers (5, 6).

Peptidoglycan is synthetized by glycosyltransferases (GTases) which catalyze the addition of a disaccharide pentapeptide precursor (lipid II) to the nascent PG chain (Fig. 1A), while transpeptidase (TPase) enzymes cross-link a proportion of the pentapeptides to adjacent PG strands (7, 8). In the absence of the bifunctional transmembrane GTase and TPase enzymes, known as class A penicillin-binding proteins (aPBPs) (Fig. 1A), Bacillus subtilis cells are viable but grow slowly, and the cells are longer and thinner (9, 10). The viability of B. subtilis lacking all aPBPs (referred to as Δ*4*) depends on the functionality of the essential protein RodA, which was recently identified to function as a PG glycosyltransferase (11, 12). The Δ*4* strain is conditionally lethal on Penassay Broth (PAB) agar medium unless the medium is supplemented with magnesium (10). It is thought that this divalent cation alters the structure of the wall by binding to teichoic acids or PG and, by doing so, might alter the activity of cell wall hydrolases (Fig. 1A), the enzymes that modify and sculpt synthesized PG to allow the cell to elongate and divide (5, 13–15).

In B. subtilis 168, both LTAs and WTAs are composed of polyglycerolphosphate (polyGroP) but are generated by independent biosynthetic pathways (Fig. 1B) (2–5). Importantly, B. subtilis is not able to survive the loss of both LTA and WTA synthesis (16), and these teichoic acids seem to have roles in both cell growth (17) and division-separation (16, 18). It has also been determined that the anionic charge of these polymers can be modified by the addition of d-alanine or *N*-acetylglucosamine (19). d-Alanylation by Dlt proteins (Fig. 1A) has been proposed to regulate autolysin activity and cell wall ion homeostasis (3, 5) and also imparts some resistance to positively charged cationic antimicrobial peptides (CAMPs) (20). The net cell envelope charge has been proposed to be moderated by the teichoic acids and the phospholipid composition of the cell membrane (Fig. 1) (21, 22). In this respect, the transmembrane protein MprF is the only enzyme known to synthesize and translocate aminoacyl-phosphatidylglycerol (aaPGol), particularly lysyl-phosphatidylglycerol (LysPGol) (Fig. 1) (23–26). MprF plays a role in the virulence of bacterial pathogens (26, 27), and in B. subtilis, it confers resistance to CAMPs (Fig. 1A) such as nisin and daptomycin (DAP) through mechanisms that are not yet characterized (28, 29). Phosphatidylglycerol (PGol) is also the substrate for LTA synthases, and in B. subtilis, three LTA synthases are present: LtaS (housekeeping), YfnI (stress), and YqgS (sporulation) (16, 18) (Fig. 1). These transmembrane LTA synthases have an extracellular catalytic domain that uses PGol to generate a polyGroP polymer on a lipid anchor (2, 3). The lipid anchor is usually a diglucosyldiacylglycerol (Glc_2_DAG) produced by UgtP on the internal surface of the cell membrane (30) (Fig. 1A). As a *ugtP* mutant still produces LTA (31), this suggests that polyGroP polymers are probably anchored to a phosphatidylglycerol, as observed in Staphylococcus aureus (32, 33).

In S. aureus, LTA is essential and seems to modulate autolytic activity (34), while WTA is dispensable but appears to restrict autolytic activity (1, 4). Contrary to B. subtilis, S. aureus has only one LTA synthase (LtaS), whose absence is (conditionally) essential (35) and can be complemented by B. subtilis *ltaS* but not *yfnI* (18, 36). A recent *in vitro* study showed that S. aureus LtaS is processive and that LTA polymer elongation is regulated directly by the identity and concentration of the lipid starter units. Interestingly, the use of Glc_2_DAG as a starter unit led to the formation of shorter polymers (33), while the use of PGol resulted in the synthesis of longer polymers. It is not clear if this newly identified mechanism is conserved in B. subtilis. However, in the absence of B. subtilis LtaS, YfnI activity results in LTA of increased length, and it is possible that the combination of LtaS and YfnI activities modulate the length of LTA (Fig. 1A) (18, 37).

By characterizing B. subtilis strains lacking peptidoglycan GTase activity (12), we aimed to understand the cell wall stresses that cause the inability of aPBP mutants to grow on glucose-rich media. Collectively, our data support the idea that in B. subtilis, the LTA length acts to moderate the activity of at least one important autolysin, LytE (Fig. 1A). The presence of glucose in a strain lacking aPBPs increases the expression of LtaS and YfnI and results in altered LTA production. Crucially, we identify a new role of MprF in altering LTA polymerization in both B. subtilis and S. aureus. Our results reveal that MprF acts to change the cell envelope in a more dramatic way than simply increasing the positive charge on the cell membrane and have important implications for understanding the association of MprF with both virulence and antibiotic resistance in Gram-positive bacteria.

## RESULTS

### MprF alters cell viability in the absence of *ponA* or *mbl*.

**FIG 2:**
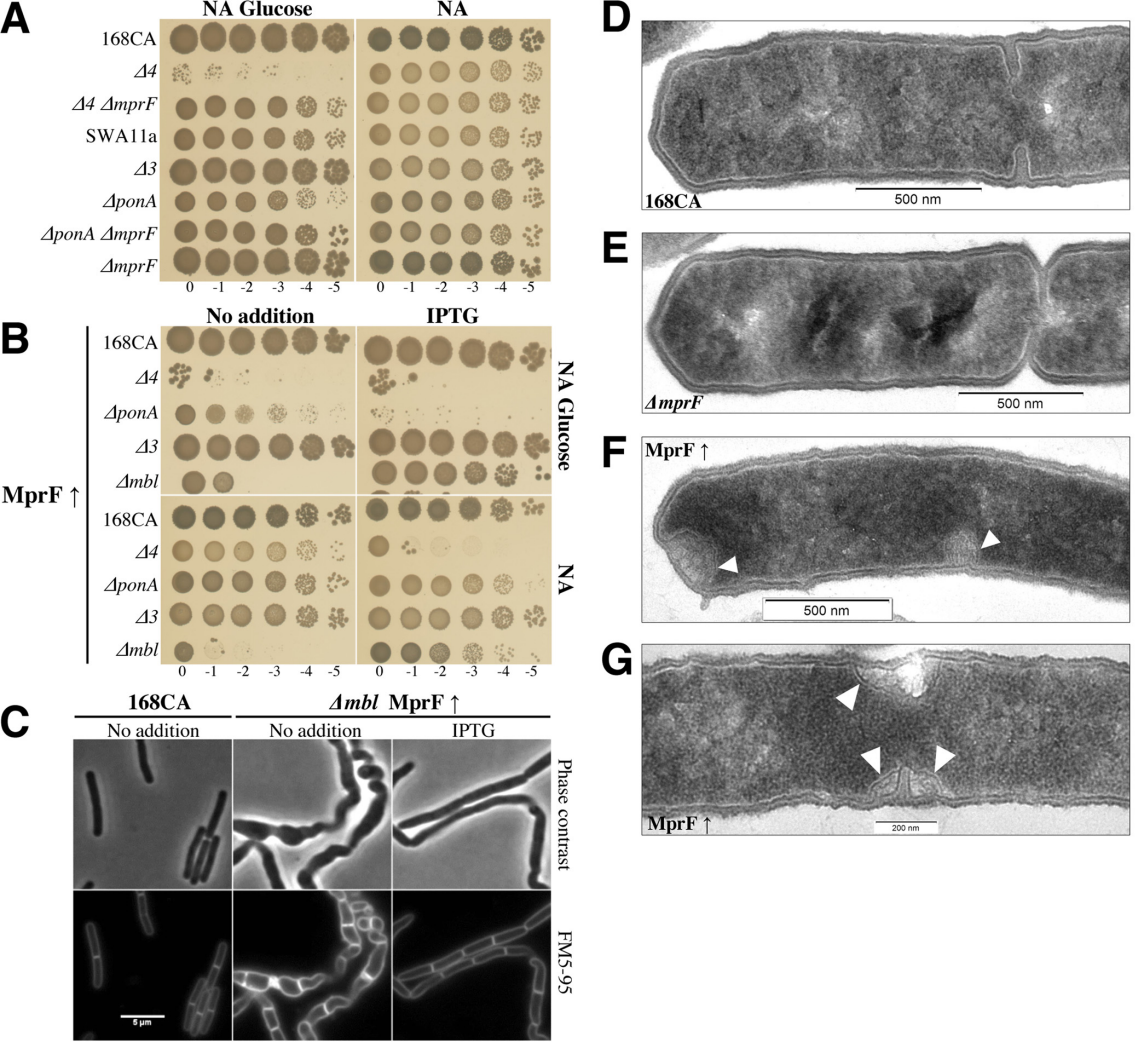
MprF alters the cell viability of B. subtilis class A PBP and *mbl* mutants. (A) Tenfold spot growth assays of B. subtilis strains 168CA (wild type), AG157 (Δ*ponA* Δ*pbpD* Δ*pbpF* Δ*pbpG* [termed Δ*4*]), AG223 (Δ*4* Δ*mprF*), SWA11a (Δ*ponA* Δ*pbpD* Δ*pbpF mprF*::Tn*YLB-1 lacA*), AG417 (Δ*pbpD* Δ*pbpF* Δ*pbpG* [termed Δ*3*]), RE101 (Δ*ponA*), AG193 (Δ*ponA* Δ*mprF*), and AG181 (Δ*mprF*). NA plates (with 1% glucose) were incubated at 37°C for 24 h, and images were taken. (B) The effect of *mprF* overexpression (MprF↑) was assayed in different strain backgrounds using an IPTG-inducible *mprF* construct. NA plates (with 1% glucose) were incubated at 37°C for 24 h and scanned. The strains tested were AG304 (168CA MprF↑), AG317 (Δ*4* MprF↑), AG311 (Δ*ponA* MprF↑), AG421 (Δ*3* MprF↑), and AG322 (Δ*mbl* MprF↑). (C) Phase-contrast and membrane dye (FM5-95) microscopy images of B. subtilis strains 168CA and AG322 (Δ*mbl* MprF↑) grown in NB with or without IPTG (0.1 mM) to mid-exponential phase at 37°C. Bar, 5 μm (D to G) Transmission electron microscopy (TEM) images showing cross sections of strains 168CA (wild type) (D), AG181 (Δ*mprF*) (E), and AG304 (MprF↑) (F and G) grown in nutrient broth (and IPTG for AG304). Arrows indicate abnormal cell wall structures that seem specific to the MprF↑ strain. All images were taken at a ×15,000 magnification and processed in Fiji with scale bars attached. Strain phenotypes were tested at least three times, and the TEM experiment was carried out one time. Supporting information for this figure is presented in Fig. S1 and Fig. S2A and B in the supplemental material.

In Bacillus subtilis, the absence of all aPBPs (Δ*ponA* Δ*pbpD* Δ*pbpF* Δ*pbpG* mutant, referred to as Δ*4* here) is conditionally lethal on glucose-rich PAB medium unless supplemented with magnesium (10, 12, 38). The suppressive effect of divalent magnesium has been observed in other cell wall-deficient mutants (10, 16, 38, 39), and it is not well understood. To identify the cell wall factors that are able to suppress the lethality of aPBP mutants, we applied random transposition mutagenesis to a strain deleted for the vegetative aPBPs (Tables 1 to 3). Mapping of the transposon in strain SWA11a revealed an insertion in the *mprF* gene, encoding a lysyl-phosphatidylglycerol (LysPGol) synthase known to participate in phospholipid metabolism (Fig. 1) (23, 28). The introduction of an *mprF* deletion into Δ*4* confirmed that the Δ*4* Δ*mprF* strain was viable on nutrient agar (NA) with glucose (NA-glucose) and PAB agar (Fig. 2A; see also Fig. S1A in the supplemental material). A strain lacking *ponA*, encoding the major aPBP (PBP1), is viable but exhibits a growth phenotype that is suppressed by magnesium (Fig. 2A and Fig. S1B). Here, the deletion of *mprF* in the Δ*ponA* strain improved its growth and colony morphology.

**TABLE 1:** Plasmids used in this study^*a*^

Plasmid	Relevant feature(s)	Reference or source
pAM-21	*kan P_T7_* LytE^(26–334)^-His_6_ *lacI*	This study
pCotC-GFP	Used as a matrix to amplify *cat*	75
pDR111	*bla amyE*::(*lacI P_hyspank_ spc*)	D. Rudner, unpublished data
pDR111-*P_hyspank_*-*ltaS*	*bla amyE*::(*lacI P_hyspank_-ltaS spc*)	This study
pDR111-*P_hyspank_*-*mprF*	*bla amyE*::(*lacI P_hyspank_-mprF spc*)*	This study
pDR111-*P_hyspank_*-*yfnI*	*bla amyE*::(*lacI P_hyspank_-yfnI spc*)	This study
pDR111-*P_hyspank_*-*yqgS*	*bla amyE*::(*lacI P_hyspank_-yqgS spc*)	This study
pET28a(+)	*kan P_T7_ his lacI*	Novagen
pG^+^host9::Δ*ponA*	*erm*, carrying flanking DNA regions of *ponA*	12
pG^+^host10::Δ*pbpD*	*bla*, carrying flanking DNA regions of *pbpD*	12
pG^+^host10::Δ*pbpF*	*bla*, carrying flanking DNA regions of *pbpF*	12
pIC22	Used as a matrix to amplify *phleo*	76
pLOSS*	*bla spc P_spac_ mcs P_divIVA_-lacZ lacI rep_pLS20_*(*GA*→*CC*)	39
pLOSS-*P_spac_*-*lytE*	*bla spc P_spac_-lytE P_divIVA_-lacZ lacI rep_pLS20_*(*GA→CC*)	This study
pLOSS-*P_spac_*-*ponA*	*bla spc P_spac_-ponA P_divIVA_-lacZ lacI rep_pLS20_*(*GA→CC*)	12
pMarB	*bla erm* Tn*YLB-1*::*kan Himar1* transposase	77
pMUTinHis	*bla erm P_spac_ mcs* His_12_ *lacZ lacI*	73
pMUTin-*′lytE-his*	*bla erm P_spac_ mcs ′*LytE-6 aa-His_12_ *lacZ lacI*	This study
pMUTin-*′ltaS-his*	*bla erm P_spac_ mcs ′*LtaS-6 aa-His_12_ *lacZ lacI*	This study
pMUTin-*′yfnI-his*	*bla erm P_spac_ mcs ′*YfnI-6 aa-His_12_ *lacZ lacI*	This study

a Plasmids are in alphabetical order. *cat*, chloramphenicol resistance; *bla*, ampicillin resistance; *spc*, spectinomycin resistance; *erm*, erythromycin resistance; *phleo*, phleomycin resistance; *kan*, kanamycin resistance; *lacI*, gene encoding a lactose repressor; *mcs*, multiple-cloning site; *lacZ*, gene encoding β-galactosidase; aa, amino acids [LEMRGS]; *rep_pLS20_*(*GA→CC*), unstable replication in *Bacillus*. *P_hyspank_* and *P_spac_* are IPTG-inducible promoters, with *P_hyspank_* being known to be stronger. *, For pDR111-*P_hyspank_*-*mprF* the native start codon was changed to ATG.

**TABLE 2:** Strains used in this study

Strain	Relevant genotype or description^*a*^	Source(s) and/or reference^*b*^
E. coli		
DH5α	F^−^ *endA1 glnV44 thi-1 recA1 relA1 gyrA96 deoR nupG* ϕ80d*lacZ*ΔM15 Δ(*lacZYA-argF*)*U169 hsdR17*(r_K_^−^ m_K_^+^) λ^−^	Lab stock
BL21(DE3)	B F^−^ *ompT gal dcm lon hsdS_B_*(r_B_^−^ m_B_^−^) λ(DE3[*lacI lacUV5*-*T7p07 ind1 sam7 nin5*]) [*malB^+^*]K-12(λ^s^)	Novagen
B. subtilis		
168CA	*trpC2*	R. Daniel, lab stock
168 1A1	*trpC2* (BGSC strain 1A1)	54
3725CA	*trpC2* Ω*neo* Δ*mreB*	3725 (78)→168CA cells
4261CA	*trpC2* Δ*mbl*::*cat*	4261 (79)→168CA cells
4285CA	*trpC2* Δ*ltaS*::*cat*	4285 (79)→168CA cells
4287CA	*trpC2* Δ*yfnI*::*spc*	4287 (79)→168CA cells
4289CA	*trpC2* Δ*yfnI*::*erm*	4289 (79)→168CA cells
4292CA	*trpC2* Δ*yqgS*::*spc*	4292 (79)→168CA cells
4293CA	*trpC2* Δ*yqgS*::*cat*	4293 (79)→168CA cells
4294CA	*trpC2* Δ*yqgS*::*erm*	4294 (79)→168CA cells
4620	*trpC2* Δ*ltaS*::*neo* Δ*yfnI*::*cat* Δ*yqgS*::*spc* Δ*yvgJ*::*erm*	79
AG105	*trpC2* Δ*pbpD* Δ*pbpF*	pG^+^host10::Δ*pbpF* (12)→RE103 cells
AG108	*trpC2* Δ*ponA* Δ*pbpD* Δ*pbpF*	pG^+^host10::Δ*pbpF*→RE104 cells
AG123	*trpC2* Δ*ponA* Δ*pbpD* Δ*pbpF* pLOSS-*P_spac_-ponA*	pLOSS-*P_spac_-ponA*→AG108 cells
AG141	*trpC2* Δ*ponA* Δ*pbpD* Δ*pbpF* pLOSS-*P_spac_-ponA lacA*::*cat*	K532→AG123 cells
AG157	*trpC2* Δ*4* (Δ*ponA* Δ*pbpD* Δ*pbpF* Δ*pbpG*::*kan*)	12
AG181	*trpC2* Δ*mprF*::*phleo*	This study
AG185	*trpC2* Δ*mprF*::*phleo* Ω*neo* Δ*mreB*	3725CA→AG181 cells
AG189	*trpC2* Δ*mprF*::*phleo* Δ*mbl*::*cat*	4261CA→AG181 cells
AG193	*trpC2* Δ*ponA* Δ*mprF*::*phleo*	AG181→RE101 cells
AG200	*trpC2* Δ*ponA* Δ*pbpD* Δ*pbpF* pLOSS-*P_spac_-ponA* Δ*mprF*::*phleo lacA*::*cat*	AG181→AG141 cells
AG200BK#42	*trpC2* Δ*ponA* Δ*pbpD* Δ*pbpF* pLOSS-*P_spac_-ponA* Δ*mprF*::*phleo lacA*::*cat gtaB*::Tn*YLB-1*(*kan*)	This study
AG210	*trpC2* Δ*4* (Δ*ponA* Δ*pbpD* Δ*pbpF* Δ*pbpG*::*kan*) Δ*mprF*::*phleo lacA*::*cat* pLOSS-*P_spac_-ponA*	DPVB45CA→AG200 cells
AG211	*trpC2* Δ*4* (Δ*ponA* Δ*pbpD* Δ*pbpF* Δ*pbpG*::*kan*) pLOSS-*P_spac_-ponA*	DPVB45CA→AG123 cells
AG223	*trpC2* Δ*4* (Δ*ponA* Δ*pbpD* Δ*pbpF* Δ*pbpG*::*kan*) Δ*mprF*::*phleo*	AG181→AG157 cells
AG229	*trpC2* Δ*4* (Δ*ponA* Δ*pbpD* Δ*pbpF* Δ*pbpG*::*kan*)	AG211 plasmid lost on 20 mM MgSO_4_ with *kan*
AG290	*trpC2* Δ*4* (Δ*ponA* Δ*pbpD* Δ*pbpF* Δ*pbpG*::*kan*) pLOSS-*P_spac_-ponA* Δ*gtaB*::*erm*	SM08→AG211 cells
AG290NopLoss	*trpC2* Δ*4* (Δ*ponA* Δ*pbpD* Δ*pbpF* Δ*pbpG*::*kan*) Δ*gtaB*::*erm*	AG290 plasmid lost on 20 mM MgSO_4_ with *kan*
AG292	*trpC2* Δ*4* (Δ*ponA* Δ*pbpD* Δ*pbpF* Δ*pbpG*::*kan*) pLOSS-*P_spac_-ponA* Ω*erm* Δ*tagE*	SM50→AG211 cells
AG293	*trpC2* Δ*4* (Δ*ponA* Δ*pbpD* Δ*pbpF* Δ*pbpG*::*kan*) *ggaAB*::*spc*	SM31→AG229 cells
AG296	*trpC2* Δ*4* (Δ*ponA* Δ*pbpD* Δ*pbpF* Δ*pbpG*::*kan*) pLOSS-*P_spac_-ponA pgcA*::*tet*	SSB122→AG211 cells
AG304	*trpC2 amyE*::(*P_hyspank_-mprF spc*)	pDR111-*P_hyspank_-mprF*→168CA cells
AG311	*trpC2* Δ*ponA amyE*::(*P_hyspank_-mprF spc*)	AG304→RE101 cells
AG317	*trpC2* Δ*4* (Δ*ponA* Δ*pbpD* Δ*pbpF* Δ*pbpG*::*kan*) *amyE*::(*P_hyspank_-mprF spc*)	AG304→AG157 cells
AG322	*trpC2 amyE*::(*P_hyspank_-mprF spc*) Δ*mbl*::*cat*	4261CA→AG304 cells
AG327	*trpC2 amyE*::(*P_hyspank_-mprF spc*) Ω*neo* Δ*mreB*	3725CA→AG304 cells
AG341	*trpC2* Δ*4* (Δ*ponA* Δ*pbpD* Δ*pbpF* Δ*pbpG*::*kan*) *dltAB*::*cat*	DLT71 (40)→AG157 cells
AG342	*trpC2* Δ*4* (Δ*ponA* Δ*pbpD* Δ*pbpF* Δ*pbpG*::*kan*) Δ*ltaS*::*cat*	4285CA→AG157 cells
AG343	*trpC2* Δ*4* (Δ*ponA* Δ*pbpD* Δ*pbpF* Δ*pbpG*::*kan*) Δ*yfnI*::*erm*	4289CA→AG157 cells
AG344	*trpC2* Δ*4* (Δ*ponA* Δ*pbpD* Δ*pbpF* Δ*pbpG*::*kan*) Δ*yqgS*::*erm*	4294CA→AG157 cells
AG347	*trpC2* Δ*mprF*::*phleo* Δ*ltaS*::*cat*	4285CA→AG181 cells
AG349	*trpC2 amyE*::(*P_hyspank_-mprF spc*) Δ*ltaS*::*cat*	4285CA→AG304 cells
AG353	*trpC2 amyE*::(*P_hyspank_-mprF spc*) Δ*yfnI*::*erm*	4289CA→AG304 cells
AG370	*trpC2* Δ*4* (Δ*ponA* Δ*pbpD* Δ*pbpF* Δ*pbpG*::*kan*) Δ*ltaS*::*cat* Δ*yfnI*::*erm*	4289CA→AG342 cells
AG372	*trpC2* Δ*4* (Δ*ponA* Δ*pbpD* Δ*pbpF* Δ*pbpG*::*kan*) Δ*ltaS*::*cat* Δ*yqgS*::*erm*	4294CA→AG342 cells
AG377	*trpC2* Δ*4* (Δ*ponA* Δ*pbpD* Δ*pbpF* Δ*pbpG*::*kan*) Δ*yqgS*::*erm* Δ*yfnI*::*spc*	4287CA→AG344 cells
AG380	*trpC2* Δ*4* (Δ*ponA* Δ*pbpD* Δ*pbpF* Δ*pbpG*::*kan*) Δ*ltaS*::*cat* Δ*yqgS*::*erm* Δ*yfnI*::*spc*	4287CA→AG372 cells
AG383	*trpC2* Δ*ponA* Δ*ltaS*::*cat*	4285CA→RE101 cells
AG384	*trpC2* Δ*ponA* Δ*yfnI*::*erm*	4289CA → RE101 cells
AG385	*trpC2* Δ*ponA* Δ*yqgS*::*spc*	4292CA→RE101 cells
AG389	*trpC2* Δ*4* (Δ*ponA* Δ*pbpD* Δ*pbpF* Δ*pbpG*::*kan*) Δ*mprF*::*phleo* Δ*ltaS*::*cat* Δ*yqgS*::*erm*	AG223→AG372 cells
AG390	*trpC2* Δ*4* (Δ*ponA* Δ*pbpD* Δ*pbpF* Δ*pbpG*::*kan*) Δ*mprF*::*phleo* Δ*ygqS*::*erm* Δ*yfnI*::*spc*	AG223→AG377 cells
AG393	*trpC2* Δ*ponA* Δ*ltaS*::*cat* Δ*yfnI*::*erm*	4289CA→AG383 cells
AG394	*trpC2* Δ*ponA* Δ*ltaS*::*cat* Δ*yqgS*::*spc*	4292CA→AG383 cells
AG395	*trpC2* Δ*ponA* Δ*yfnI*::*spc* Δ*yqgS*::*spc*	4292CA→AG384 cells
AG399	*trpC2* Δ*4* (Δ*ponA* Δ*pbpD* Δ*pbpF* Δ*pbpG*::*kan*) Δ*mprF*::*phleo* Δ*ltaS*::*cat* Δ*yfnI*::*erm*	AG223→AG370 cells
AG400	*trpC2* Δ*4* (Δ*ponA* Δ*pbpD* Δ*pbpF* Δ*pbpG*::*kan*) Δ*mprF*::*phleo* Δ*ltaS*::*cat* Δ*yqgS*::*erm* Δ*yfnI*::*spc*	AG223→AG380 cells
AG403	*trpC2* Δ*ponA* Δ*ltaS*::*cat* Δ*yfnI*::*erm* Δ*yqgS*::*spc*	4292CA→AG393 cells
AG417	*trpC2* Δ*3* (Δ*pbpD* Δ*pbpF* Δ*pbpG*::*kan*)	DPVB45CA→AG105 cells
AG421	*trpC2* Δ*3* (Δ*pbpD* Δ*pbpF* Δ*pbpG*::*kan*) *amyE*::(*P_hyspank_-mprF spc*)	AG304→AG417 cells
AG443	*trpC2* Δ*4* (Δ*ponA* Δ*pbpD* Δ*pbpF* Δ*pbpG*::*kan*) Δ*ugtP*::*spc*	PG253→AG157 cells
AG444	*trpC2* Δ*ponA* Δ*ugtP*::*spc*	PG253→RE101 cells
AG474	*trpC2* Δ*cwlO*::*spc*	PDC463 (43)→168CA cells
AG475	*trpC2 amyE*::(*P_hyspank_-lytE spc*)	(P. Dominguez-Cuevas, unpublished strain)→168CA cells
AG484	*trpC2* Δ*ponA amyE*::(*P_hyspank_-lytE spc*)	AG475→RE101 cells
AG501	*trpC2* Δ*4* (Δ*ponA* Δ*pbpD* Δ*pbpF* Δ*pbpG*::*kan*) *amyE*::(*P_hyspank_-lytE spc*)	AG475→AG157 cells
AG502	*trpC2* Δ*4* (Δ*ponA* Δ*pbpD* Δ*pbpF* Δ*pbpG*::*kan*) Δ*mprF*::*phleo amyE*::(*P_hyspank_-lytE spc*)	AG475→AG223 cells
AG506	*trpC2* Δ*ponA* Δ*gtaB*::*erm*	SM08→RE101 cells
AG547	*trpC2* Δ*lytE*::*cat*	This study
AG549	*trpC2* Δ*lytE*::*cat* pLOSS-*P_spac_-lytE*	pLOSS-*P_spac_-lytE*→AG547 cells
AG551	*trpC2* pLOSS-*P_spac_-lytE*	pLOSS-*P_spac_-lytE*→168CA cells
AG557	*trpC2* Δ*ponA* pLOSS-*P_spac_-lytE*	pLOSS-*P_spac_-lytE*→RE101 cells
AG565	*trpC2* Ω*lytE*-His_12_::(pMUTin-*′lytE erm*)	pMUTinHis-*'lytE*→168CA cells
AG569	*trpC2* Ω*ltaS-*His_12_::(pMUTin-′*ltaS erm*)	pMUTinHis-*'ltaS*→168CA cells
AG570	*trpC2* Ω*yfnI-*His_12_::(pMUTin-′*yfnI erm*)	pMUTinHis-*'yfnI*→168CA cells
AG586	*trpC2* Δ*cwlO*::*spc* Ω*lytE-*His_12_::(pMUTin-*′lytE erm*)	pMUTinHis-*'lytE*→AG474 cells
AG587	*trpC2* Δ*ponA* Ω*lytE-*His_12_::(pMUTin-*′lytE erm*)	AG565→RE101 cells
AG588	*trpC2* Δ*ponA* Ω*ltaS-*His_12_::(pMUTin*-′ltaS erm*)	AG569→RE101 cells
AG589	*trpC2* Δ*ponA* Ω*yfnI-*His_12_::(pMUTin*-′yfnI erm*)	AG570→RE101 cells
AG593	*trpC2* Δ*yqgS*::*spc* Δ*ltaS*::*cat*	4292CA→4285CA cells
AG594	*trpC2* Δ*yfnI*::*erm* Δ*ltaS*::*cat*	4289CA→4285CA cells
AG595	*trpC2* Δ*yfnI*::*erm* Δ*yqgS*::*spc*	4292CA→4289CA cells
AG600	*trpC2* Δ*ltaS*::*cat* Δ*yfnI*::*erm* Δ*yqgS*::*spc*	4285CA→AG595 cells
AG614	*trpC2* Δ*4* (Δ*ponA* Δ*pbpD* Δ*pbpF* Δ*pbpG*::*kan*) *lacA*::*cat* Δ*mprF*::*phleo* pLOSS-*P_spac_-ponA* Δ*gtaB*::*erm*	SM08→AG210 cells
AG632	*trpC2* Δ*4* (Δ*ponA* Δ*pbpD* Δ*pbpF* Δ*pbpG*::*kan*) Δ*mprF*::*phleo* Δ*ugtP*::*spc*	PG253→AG223 cells
AG636	*trpC2* Δ*4* (Δ*ponA* Δ*pbpD* Δ*pbpF* Δ*pbpG*::*kan*) *lacA*::*cat* Δ*mprF*::*phleo* Δ*gtaB*::*erm*	AG614 plasmid lost on NA–20 mM MgSO_4_ with *erm*
AG1460	*trpC2* Δ*3* (Δ*pbpD* Δ*pbpF* Δ*pbpG*::*kan*) *amyE*::(*P_hyspank_-lytE spc*)	AG475→AG417 cells
AG1461	*trpC2* Δ*lytE*::*cat amyE*::(*P_hyspank_-lytE spc*)	AG475→AG547 cells
AG1462	*trpC2* Δ*ltaS*::*cat amyE*::(*P_hyspank_-lytE spc*)	AG475→4285CA cells
AG1463	*trpC2* Δ*yfnI*::*erm amyE*::(*P_hyspank_-lytE spc*)	AG475→4289CA cells
AG1465	*trpC2* Δ*4* (Δ*ponA* Δ*pbpD* Δ*pbpF* Δ*pbpG*::*kan*) Δ*ltaS*::*cat amyE*::(*P_hyspank_-lytE spc*)	AG475→AG342 cells
AG1466	*trpC2* Δ*ltaS*::*cat* pLOSS-*P_spac_-lytE*	pLOSS-*P_spac_-lytE*→4285CA cells
AG1467	*trpC2* Δ*yfnI*::*erm* pLOSS-*P_spac_-lytE*	pLOSS-*P_spac_-lytE*→4289CA cells
AG1469	*trpC2* Δ*ltaS*::*cat* Δ*yfnI*::*erm* pLOSS-*P_spac_-lytE*	pLOSS-*P_spac_-lytE*→AG594 cells
AG1478	*trpC2* Δ*mprF*::*phleo amyE*::(*P_hyspank_-lytE spc*)	AG475→AG181 cells
AG1479	*trpC2* Δ*ponA* Δ*mprF*::*phleo amyE*::(*P_hyspank_-lytE spc*)	AG475→AG193 cells
AG1480	*trpC2* Δ*ponA* Δ*ltaS*::*cat amyE*::(*P_hyspank_-lytE spc*)	AG475→AG383 cells
AG1481	*trpC2* Δ*mprF*::*phleo* pLOSS-*P_spac_-lytE*	pLOSS-*P_spac_-lytE*→AG181 cells
AG1482	*trpC2* Δ*ponA* Δ*mprF*::*phleo* pLOSS-*P_spac_-lytE*	pLOSS-*P_spac_-lytE*→AG193 cells
AG1483	*trpC2* Δ*ponA* Δ*ltaS*::*cat* pLOSS-*P_spac_-lytE*	pLOSS-*P_spac_-lytE*→AG383 cells
AG1486	*trpC2* Δ*mprF*::*phleo* Ω*lytE-*His_12_::(pMUTin-*′lytE erm*)	AG565→AG181 cells
AG1487	*trpC2* Δ*ponA* Δ*mprF*::*phleo* Ω*lytE-*His_12_::(pMUTin-*′lytE erm*)	AG565→AG193 cells
AG1488	*trpC2* Δ*ponA* Δ*ltaS*::*cat* Ω*lytE-*His_12_::(pMUTin-*′lytE erm*)	AG565→AG383 cells
AG1489	*trpC2* Δ*ltaS*::*cat* Ω*lytE-*His_12_::(pMUTin-*′lytE erm*)	AG565→4285CA cells
AG1490	*trpC2* Δ*ltaS*::*cat* Δ*yqgS*::*erm*	4294CA→4285CA cells
AG1491	*trpC2* Δ*yqgS*::*cat* Δ*yfnI*::*erm*	4293CA→4289CA cells
AG1492	*trpC2* Δ*yqgS*::*cat amyE*::(*P_hyspank_-lytE spc*)	AG475→4293CA cells
AG1493	*trpC2* Δ*yqgS*::*cat* pLOSS-*P_spac_-lytE*	pLOSS-*P_spac_-lytE*→4293CA cells
AG1497	*trpC2* Δ*ltaS*::*cat* Δ*yfnI*::*erm amyE*::(*P_hyspank_-lytE spc*)	AG475→AG594 cells
AG1498	*trpC2* Δ*ltaS*::*cat* Δ*yqgS*::*erm amyE*::(*P_hyspank_-lytE spc*)	AG475→AG1490 cells
AG1499	*trpC2* Δ*yqgS*::*cat* Δ*yfnI*::*erm amyE*::(*P_hyspank_-lytE spc*)	AG475→AG1491 cells
AG1500	*trpC2* Δ*ltaS*::*cat* Δ*yqgS*::*erm* pLOSS-*P_spac_-lytE*	pLOSS-*P_spac_-lytE*→AG1490 cells
AG1502	*trpC2* Δ*yqgS*::*cat* Δ*yfnI*::*erm* pLOSS-*P_spac_-lytE*	pLOSS-*P_spac_-lytE*→AG1491 cells
AG1535	*trpC2* Δ*ugtP*::*spc* Ω*lytE-*His_12_::(pMUTin-*′lytE erm*)	AG565→PG253 cells
AG1541	*trpC2* Δ*cwlO*::*spc* Ω*lytE-*His_12_::(pMUTin-*′lytE erm*)	AG565→AG474 cells
AG1581	*trpC2* Δ*mbl*::*zeo amyE*::(*P_hyspank_-mprF spc*)	AK045B→AG304 cells
AG1593	*trpC2* Δ*mreBH*::*kan*	BGSC strain^*c*^→168CA cells
AG1594	*trpC2* Δ*ugtP*::*kan*	BGSC strain^*c*^→168CA cells
AG1604	*trpC2* Δ*ponA* Ω*neo* Δ*mreB*	KS36 → RE101 cells
AG1605	*trpC2* Δ*ponA* Δ*mbl*::*zeo*	AK045B→RE101 cells
AG1643	*trpC2* Δ*ugtP*::*kan amyE*::(*P_hyspank_-lytE spc*)	AG1594→AG475 cells
AG1663	*trpC2* Δ*mprF*::*kan*	BGSC strain^*c*^→168CA cells
AG1667	*trpC2* Δ*ltaS*::*cat* Δ*yfnI*::*erm amyE*::(*P_hyspank_-lytE spc*) Δ*mprF*::*kan*	AG1663→AG1497 cells
AG1668	*trpC2* Δ*ltaS*::*cat* Δ*yqgS*::*erm amyE*::(*P_hyspank_-lytE spc*) Δ*mprF*::*kan*	AG1663→AG1498 cells
AG1669	*trpC2* Δ*yqgS*::*cat* Δ*yfnI*::*erm amyE*::(*P_hyspank_-lytE spc*) Δ*mprF*::*kan*	AG1663→AG1499 cells
AG1670	*trpC2* Δ*ltaS*::*cat* Δ*yfnI*::*erm amyE*::(*P_hyspank_-lytE spc*) Δ*ugtP*::*kan*	AG1594→AG1497 cells
AG1671	*trpC2* Δ*ltaS*::*cat* Δ*yqgS*::*erm amyE*::(*P_hyspank_-lytE spc*) Δ*ugtP*::*kan*	AG1594→AG1498 cells
AG1672	*trpC2* Δ*yqgS*::*cat* Δ*yfnI*::*erm amyE*::(*P_hyspank_-lytE spc*) Δ*ugtP*::*kan*	AG1594→AG1499 cells
AG1673	*trpC2* Δ*ltaS*::*cat* Δ*yqgS*::*spc* Δ*mprF*::*kan*	AG1663→AG593 cells
AG1675	*trpC2* Δ*yfnI*::*erm* Δ*yqgS*::*spc* Δ*mprF*::*kan*	AG1663→AG595 cells
AG1684	*trpC2* Δ*ponA* Ω*lytE-his_12_*::(pMUTin-*′lytE erm*) *amyE*::(*P_hyspank_-mprF spc*)	AG304→AG587 cells
AG1685	*trpC2* Δ*ponA* Ω*lytE-*His_12_::(pMUTin-*′lytE erm*) Δ*ugtP*::*kan*	AG1594→AG587 cells
AK045B	*trpC2* Δ*mbl*::*zeo* (168CA background)	H. Strahl’s lab, A. Koh
Dap^R^1	DAP-resistant isolate derived from 168 1A1	54
Dap^R^20	DAP-resistant isolate derived from 168 1A1	54
DLT71CA	*trpC2 dltAB*::*cat*	DLT71 (40)→168CA cells
DPVB45CA	*trpC2* Δ*pbpG*::*kan*	DPVB45 (80)→168CA
HB15507	Dap^R^20 *pgsA*^WT^	54
HB15516	Dap^R^1 *pgsA*^WT^	54
K532	*trpC2 lacA*::*cat*	D. Claessen, unpublished data
KS36	*trpC2* Ω*neo* Δ*mreB*	H. Strahl’s lab, K. Seistrup
PG253	*trpC2* Δ*ugtP*::*spc*	P. Gamba, unpublished data
RE101	*trpC2* Δ*ponA*	12
RE102	*trpC2* Δ*pbpF*	pG^+^host10::Δ*pbpF* (12)→168CA cells
RE103	*trpC2* Δ*pbpD*	pG^+^host10::Δ*pbpD* (12)→168CA cells
RE104	*trpC2* Δ*ponA* Δ*pbpD*	pG^+^host9::Δ*ponA* (12)→RE103 cells
SM08	*trpC2* Δ*gtaB*::*erm*	S. Moore, unpublished data
SM31	*trpC2 ggaAB*::*spc*	S. Moore, unpublished
SM50	*trpC2* Ω*erm* Δ*tagE*	S. Moore, unpublished
SSB122	*trpC2 pgcA* (*yhxB*)::*tet*	81
SWA11a	*trpC2* Δ*ponA* Δ*pbpD* Δ*pbpF lacA*::*cat mprF*::Tn*YLB-1*(*kan*)	This study
S. aureus		
SA113	SA113 (wild type, methicillin sensitive)	T. Palmer, lab collection
SA113 Δ*mprF*	SA113 Δ*mprF*::*erm*	21

a *spc*, antibiotic resistance to spectinomycin; *kan*, antibiotic resistance to kanamycin; *erm*, antibiotic resistance to erythromycin; *neo*, antibiotic resistance to neomycin; *cat*, antibiotic resistance to chloramphenicol; *tet*, antibiotic resistance to tetracycline; *zeo*, antibiotic resistance to zeocin; *phleo*, antibiotic resistance to phleomycin. Tn*YLB-1*(*kan*) is a transposon that originates from pMarB (Table 1). His_12_ indicates a coding sequence that corresponds to a His_12_ tag at the C terminus. *P_hyspank_* and *P_spac_* are IPTG-inducible promoters, with *P_hyspank_* being known to be stronger. Δ indicates a deletion, and Ω indicates an insertion. In this study, the mutations Δ*ponA* Δ*pbpD* Δ*pbpF* Δ*pbpG*::*kan* and Δ*pbpD* Δ*pbpF* Δ*pbpG*::*kan* have the simplified annotations Δ*4* and Δ*3*, respectively. Strains KS36, PG253, SM08, SM31 and SM50 were made in the 168CA background.

b Arrows indicate how the strain was constructed by transformation using either plasmid DNA or chromosomal DNA (the strain name is then indicated).

c BGSC refers to the deletion mutant collection of the *Bacillus* Genetic Stock Center (82).

**TABLE 3:** Primers used in this study

Primer name	Sequence^*a*^	Use(s)^*b*^
oAA01	TTTTTCACCTTAATGCTTTGCATGGTATATCTCCTTCTTAAAGTTAAACAAAATTATTTCTAGAGG	Amplification of pET28a(+)
oAA02	ACCTCGGTGCGAAAAGATTCCACCACCACCACCACCACT	Amplification of pET28a(+)
oAA03	TAAGAAGGAGATATACCATGCAAAGCATTAAGGTGAAAAAAGGCGAC	Amplification of *lytE* from bases 76–1002
oAA04	CAGTGGTGGTGGTGGTGGTGGAATCTTTTCGCACCGAGGTAACG	Amplification of *lytE* from bases 76–1002
oAA05	ACGCCGAAACAAGCGCTC	Check the pET28a-*lytE-his* plasmid
oAA06	GCTAGTTATTGCTCAGCG	Check the pET28a-*lytE-his* plasmid
oAG18	GTTGACTTTATCTACAAGGT	Check the insert cloned into pLOSS-*P_spac_*-*lytE*
oAG19	GGTACCAGTAGTTCACCAC	Check the insert cloned into pLOSS-*P_spac_*-*lytE*
oAG81	GAATCGCCTGGCTCACATCATC	Check B. subtilis *mprF*::Tn*YLB-1*
oAG82	CGGCAAAGAACAGTCCCAAG	Check B. subtilis *mprF*::Tn*YLB-1*
oAG103	CCTGTATGTCTGGCACTCAC	Amplification upstream of *mprF*
oAG104	GC**TCTAGA**TGGTCTCTCCAATCATATTC	Amplification upstream of *mprF* (*Eco*RI)
oAG105	CG**GAATTC**TAAGACGGAGTCTTTTTTTATTTCG	Amplification downstream of *mprF* (*Xba*I)
oAG106	CGGAGATGCGAGAGGGTTTC	Amplification downstream of *mprF*
oAG107	TGCGGCATGCTCATATCCAG	Check B. subtilis Δ*mprF*
oAG108	TGGGCCAATCAGTGGACAAG	Check B. subtilis Δ*mprF*
oAG122	GCGTCTGCCTGAAATTAACC	Check B. subtilis Δ*mbl*
oAG123	GCAATCATTGCGGATGTTGC	Check B. subtilis Δ*mbl*
oAG124	CCATCATCTGGTGCGAAAGG	Check B. subtilis Δ*ponA*^*c*^
oAG125	CCGCAAAGCCGATTAATTGG	
oAG126	TCTATTGGCGAGTGCTTC	Check B. subtilis Δ*pbpF*^*c*^
oAG127	AGCATCGACTCCGTATTG	
oAG128	TTATTCGGAATGGCGATGGG	Check B. subtilis Δ*pbpD*^*c*^
oAG129	CCTTAATGGCTGCAGTTGAC	
oAG165	CTAACCTGGCTGACATTCAC	Check B. subtilis *gtaB*::Tn*YLB-1*
oAG166	TGATCAGGTCTTCGCAGTTG	Check B. subtilis *gtaB*::Tn*YLB-1*
oAG197	GC**TCTAGA**GCACCCATTAGTTCAACAAACG	Amplification of *cat* (XbaI)
oAG198	GC**GGATCC**AGTACAGTCGGCATTATCTC	Amplification of *cat* (BamHI)
oAG261	GCCAGTGAATCGCTGGAAAG	Check B. subtilis Δ*ugtP*
oAG262	ATCTGGGAGCACCCGTCAAG	Check B. subtilis Δ*ugtP*
oAG271	GGG**GTCGAC**AAAGGAGATTCCTAGGATGCTGATTAAAAAGAATGCT	Construction of pDR111-*P_hyspank_*-*mprF*
oAG272	GGG**GCATGC**TTGATGATATTGAAACCT	Construction of pDR111-*P_hyspank_*-*mprF*
oAG300	CAATCACGAAACAATAATTGG	Check the insert cloned into pDR111
oAG301	GTTGACTTTATCTACAAGGTG	Check the insert cloned into pDR111
oAG308	GGG**GCTAGC**AAAGGAGATTCCTAGGATGAAAACATTTATAAAAGAAAGAGG	Construction of pDR111-*P_hyspank_*-*ltaS* (*Nhe*I)
oAG309	CCG**GCATGC**CCGAATGTGGAATTTGC	Construction of pDR111-*P_hyspank_*-*ltaS* (*Sph*I)
oAG310	GGG**GCTAGC**AAAGGAGATTCCTAGGATGAAGAAACTTTTTTCTTACAAAC	Construction of pDR111-*P_hyspank_*-*yfnI* (*Nhe*I)
oAG311	CCG**GCATGC**TGTAATGATATGAGAGAAAGC	Construction of pDR111-*P_hyspank_*-*yfnI* (*Sph*I)
oAG312	GGG**GCTAGC**AAAGGAGATTCCTAGGATGCGAAAAACGTTTTTTTCGAAG	Construction of pDR111-*P_hyspank_*-*yqgS* (*Nhe*I)
oAG313	CCGGCATGCGAAAGCCTCCCGCTCACTTC	Construction of pDR111-*P_hyspank_*-*yqgS* (*Sph*I)
oAG314	TCGTCCAGTGATTGGTTTCC	Check B. subtilis Δ*yfnI*
oAG315	GACCGCTTTCATCTCTACCC	Check B. subtilis Δ*yfnI*
oAG316	CGCCACTTTCTCCCTCATAC	Check B. subtilis Δ*ltaS*
oAG317	GTCAAATCGGGCGGGCAATC	Check B. subtilis Δ*ltaS*
oAG318	CGTATCGAGAGCCGGAGAAC	Check B. subtilis Δ*yqgS*
oAG319	GAAGCTCTTTGCCGCTATGC	Check B. subtilis Δ*yqgS*
oAG340	GTCACATGAAGTCAAGACTATT	Check B. subtilis Δ*lytE*
oAG341	ACGGTTTATCAAGGAAGGACTC	Check B. subtilis Δ*lytE*
oAG348	GTG**GCGGCCGC**ATGAAAAAGCAAATCATTACAG	Construction of pLOSS-*P_spac_*-*lytE* (*Not*I)
oAG349	CCG**GGATCC**GATTGCCCTTTATGAAAATAAG	Construction of pLOSS-*P_spac_*-*lytE* (*Bam*HI)
oAG350	GTCTGTGCTTGAGGATAAGG	Amplification upstream of *lytE* for construction of Δ*lytE*
oAG351	GGG**TCTAGA**CTGCTGTCGTAGCTGTAATG	Amplification upstream of *lytE* (XbaI) for construction of Δ*lytE*
oAG352	CCG**GGATCC**GCGAAAAGATTCTAATTTTTAG	Amplification downstream of *lytE* (BamHI) for construction of Δ*lytE*
oAG353	GATCCGTTTGCGTGTTTC	Amplification downstream of *lytE* for construction of Δ*lytE*
oAG354	GGG**GAATTC**GGCGGAACGACAACTTCAG	Construction of pMUTin-′*lytE-his*, amplification of the 3′ terminus of *′lytE* (no stop codon)
oAG355	GGG**CTCGAG**GAATCTTTTCGCACCGAGG	Construction of pMUTin-*′lytE-his*, amplification of the 3′ terminus of *′lytE* (no stop codon)
oAG360	GGC**GAATTC**GATTTATTCTCTAAAGACCACG	Construction of pMUTin-*′yfnI-his*, amplification of the 3′ terminus of *′yfnI* (no stop codon)
oAG361	GGG**CTCGAG**TTTGATTTCTTTCTCCTTGCCG	Construction of pMUTin-*′yfnI-his*, amplification of the 3′ terminus of *′yfnI* (no stop codon)
oAG366	GGC**GAATTC**CGAAACGGAGACTTTATTTCAC	Construction of pMUTin-*′ltaS-his*, amplification of the 3′ terminus of *′ltaS* (no stop codon)
oAG367	GGG**CTCGAG**TTTATCTTCGTTATCCTTTGAC	Construction of pMUTin-*′ltaS-his*, amplification of the 3′ terminus of *′ltaS* (no stop codon)
oAG368	CAAGGTGTGGCATAATGT	Check integration into pMUTinHis
oAG369	GACATCCAGAGGCACTTC	Check integration in pMUTinHis
oAG412	CACCTGGTTTTCTGCTATAGT	Check B. subtilis Δ*gtaB*
oAG413	GTTTAACTCTACAATCAGTG	Check B. subtilis Δ*gtaB*
PhleoXbaI-fw	GC**TCTAGA**TCTTCCTTCAGGTTATGAC	Amplification of *phleo* from pIC22 (*Xba*I)
PhleoEcoRI-rev	C**GAATTC**CGCGCCCGATTGCTGAAC	Amplification of *phleo* from pIC22 (*Eco*RI)

a Restriction enzyme sites are in boldface type.

b *cat*, chloramphenicol resistance; *phleo*, phleomycin resistance cassette. *P_spac_* and *P_hyspank_* are promoters inducible with IPTG, the latter of which is known to be stronger. Tn*YLB-1* is a transposon associated with kanamycin resistance.

c See reference 12.

10.1128/mbio.02667-22.1 FIG S1 Conditional growth of mutants of cell envelope components. (A) Tenfold spot dilution growth assays of B. subtilis strains 168CA (wild type), AG157 (Δ*ponA* Δ*pbpD* Δ*pbpF* Δ*pbpG* [named Δ*4*]), AG223 (Δ*4* Δ*mprF*), SWA11a (Δ*ponA* Δ*pbpD* Δ*pbpF mprF*::Tn*YLB-1 lacA*), AG417 (Δ*pbpD* Δ*pbpF* Δ*pbpG* [named Δ*3*]), RE101 (Δ*ponA*), AG193 (Δ*ponA* Δ*mprF*), and AG181 (Δ*mprF*). Serial dilutions were spotted onto nutrient agar (NA) with or without glucose (1%) (Fig. 2A) as well as onto PAB agar (this figure). Plates were incubated at 37°C for 24 h and imaged. (B) Comparison of the Δ*4* and Δ*ponA* mutants in the absence of MprF or LtaS. Strains were streaked from glycerol stocks onto NA plates (supplemented with antibiotics and/or magnesium where required). After incubation at 37°C overnight, a single colony of each strain was then streaked across the set of plates shown at the top (with 0.5% glucose and 10 mM MgSO_4_) and scanned after 16 h of incubation at 37°C. The following strains were inoculated: RE101 (Δ*ponA*), AG193 (Δ*ponA* Δ*mprF*), AG383 (Δ*ponA* Δ*ltaS*), AG157 (Δ*4*), AG223 (Δ*4* Δ*mprF*), AG342 (Δ*4* Δ*ltaS*), AG181 (Δ*mprF*), 4285CA (Δ*ltaS*), and AG347 (Δ*mprF* Δ*ltaS*). (C) Cultures of strains grown overnight were diluted back into NB and grown at 37°C in the presence or absence of IPTG (dashed or solid lines, respectively). Growth curves of strains carrying *P_hyspank_-mprF* are displayed as follows: black diamonds, wild-type-like MprF↑ (AG304 strain); red circles, Δ*ponA* MprF↑ (AG311 strain); blue triangles, Δ*4* MprF↑ (AG317 strain); green squares, Δ*mbl* MprF↑ (AG322 strain). Download FIG S1, TIF file, 1.9 MB. Copyright © 2023 Guyet et al. 2023 Guyet et al. https://creativecommons.org/licenses/by/4.0/ This content is distributed under the terms of the Creative Commons Attribution 4.0 International license.

10.1128/mbio.02667-22.2 FIG S2 Conditional growth of strains with magnesium dependence. (A and B) Growth of actin-like mutants in the 168CA background and in the absence of *ponA*. Strains were streaked from a glycerol stock onto NA plates supplemented with antibiotics and/or magnesium where required. After incubation at 37°C overnight, a single colony of each strain was then streaked across the set of plates (0.5% glucose) shown at the top and scanned after 24 h or 48 h of incubation at 37°C. The following strains obtained in the 168CA background were inoculated: Δ*mreB* (KS36), Δ*mbl* (AK045B), and Δ*mreBH* (AG1593) (A) and Δ*ponA* (RE101), Δ*ponA* Δ*mbl* (AG1605), and Δ*ponA* Δ*mreB* (AG1604) (B). (C) The absence of *gtaB* or *ugtP* causes severe growth defects in the absence of *ponA*. The Δ*ponA* (RE101), Δ*ugtP* (PG253), Δ*ponA* Δ*ugtP* (AG444), and Δ*ponA* Δ*gtaB* (AG506) strains were prepared as described above for panels A and B and then streaked across a set of plates as shown at the top. Pictures were taken after 24 h and 72 h of incubation at 37°C. Download FIG S2, TIF file, 2.7 MB. Copyright © 2023 Guyet et al. 2023 Guyet et al. https://creativecommons.org/licenses/by/4.0/ This content is distributed under the terms of the Creative Commons Attribution 4.0 International license.

In light of these results, we reasoned that if MprF activity is detrimental to the Δ*4* mutant, its overexpression should be deleterious to the Δ*ponA* mutant. Consistent with this idea, the overexpression of MprF (MprF↑) was found to be lethal in *ponA-*deleted strains on NA-glucose (Fig. 2B). In contrast, on NA, both the Δ*ponA* and Δ*4* strains grew slowly when *mprF* overexpression was induced (Fig. 2B), but no obvious effect was observed for the 168CA or Δ*3* (Δ*pbpD* Δ*pbpF* Δ*pbpG*) strain. We also observed that the overexpression of *mprF* caused Δ*ponA* and Δ*4* cells to lyse in nutrient broth (NB) (Fig. S1C).

The identification of Δ*mprF* as a suppressor mutation suggested that an increased negative charge on the membrane could contribute to the rescue of Δ*4* growth. However, this seemed to contradict the long-standing hypothetical role of magnesium in altering the cell envelope by neutralizing anionic polymers and/or autolysis activity (1, 5). As it is known that the function of the *dlt* operon is to add positive charges to LTA, it would be predicted that the loss of Dlt’s function should have a suppression effect on Δ*4* strain viability similar to that of the Δ*mprF* mutation (40, 41). However, the Δ*4* Δ*dltAB* strain was unable to grow on glucose-rich media (data not shown). We also deleted the genes encoding the cardiolipin synthases in the Δ*4* mutant to alter the membrane charge and the cellular pool of PGol (Fig. 1B), but the loss of cardiolipin also had no suppressive effect on the Δ*4* phenotype (data not shown).

To understand if the effect of MprF was specific to the Δ*4* and Δ*ponA* mutants, we altered MprF expression in other known magnesium-dependent mutants, Δ*mreB* and Δ*mbl*. It should, however, be noted that only the Δ*mbl* strain requires magnesium to be able to grow on an NA plate compared to the Δ*mreB* strain (Fig. S2A). MreB, Mbl, and MreBH are actin-like isologues that help maintain cell shape by controlling both cell wall synthesis (42) and the major autolytic enzymes CwlO and LytE (Mbl for CwlO and MreB/MreBH for LytE) (43). Interestingly, we identified that MprF overexpression rescued the growth of the Δ*mbl* strain on NA-glucose and NA (Fig. 2B). In addition, the morphological defects associated with this mutation (16) were less pronounced, resulting in wide rod-shaped cells and cell chains that were less often twisted (Fig. 2C).

The suppression of Δ*mbl* lethality by MprF overexpression raised the possibility that MprF plays a role in cell wall metabolism. Interestingly, transmission electron microscopy (TEM) imaging of a strain overexpressing MprF (Fig. 2F and G) showed an increase in cell wall thickness (and cell poles appeared “dented”) and misplaced division septa, consistent with roles in cell wall metabolism and, perhaps, cell division. However, the Δ*mprF* mutant did not show significant changes in cell morphology (Fig. 2E).

Previous reports showed that the lethality of B. subtilis Δ*mbl* is suppressed by the deletion of the major LTA synthase LtaS (16) and that in an *ltaS* mutant, the activity of the LTA synthase YfnI results in longer LTA polymers (18). This suggested that the overexpression of MprF in the Δ*mbl* mutant might diminish the cellular level of PGol (through a different pathway [Fig. 1B]) (24) or that MprF functions in LTA synthesis.

### Conditional essentiality of UgtP in the absence of MprF and the class A PBPs.

**FIG 3:**
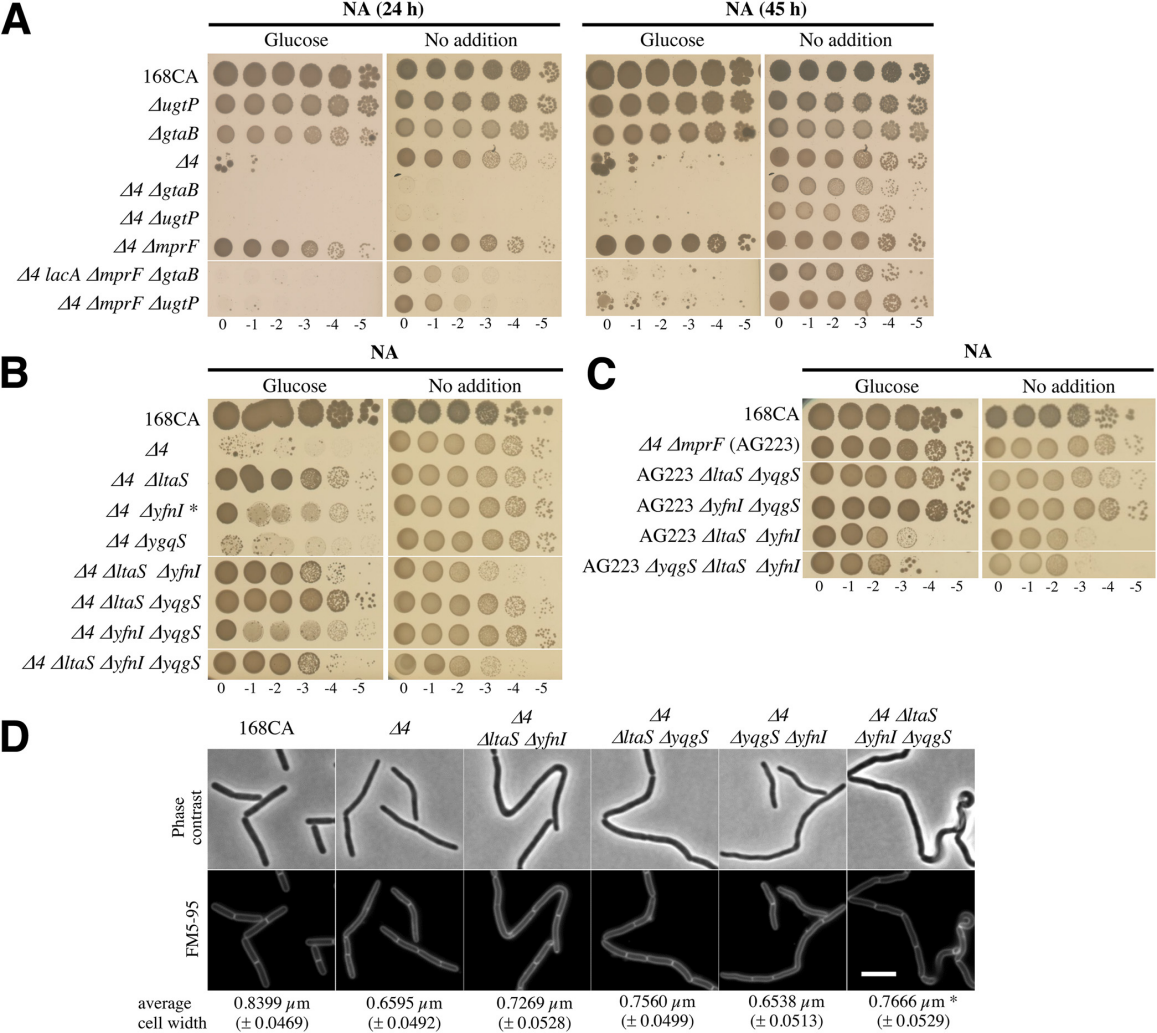
LtaS causes cell morphogenesis defects in an all-class A PBP mutant. (A) The absence of *gtaB* or *ugtP* causes a severe growth defect in the absence of class A PBPs and MprF. Strains were grown exponentially in NB with 20 mM MgSO_4_, washed, and diluted in NB prior to the preparation of a tenfold 1 serial dilution in NB with spotting onto the same NA and NA-glucose (0.5%) plates, which were incubated at 37°C and imaged after 24 h and 45 h. Strains were also spotted onto the same media supplemented with 10 mM MgSO_4_ to confirm that all of the strains’ dilution spots could grow; at 17 h of incubation, only the Δ*4* Δ*gtaB* and Δ*4* Δ*ugtP* strains displayed colonies of smaller sizes at that time point (data not shown). The strains tested were B. subtilis 168CA (wild type), PG253 (Δ*ugtP*), SM08 (Δ*gtaB*), AG157 (Δ*4*), AG290NopLoss (Δ*4* Δ*gtaB*), AG443 (Δ*4* Δ*ugtP*), AG223 (Δ*4* Δ*mprF*), AG636 (Δ*4 lacA* Δ*mprF* Δ*gtaB*), and AG632 (Δ*4* Δ*mprF* Δ*ugtP*). (B) Spot dilution growth assays for B. subtilis strains 168CA (wild type), AG157 (Δ*4*), AG342 (Δ*4* Δ*ltaS*), AG343 (Δ*4* Δ*yfnI*), AG344 (Δ*4* Δ*yqgS*), AG370 (Δ*4* Δ*ltaS* Δ*yfnI*), AG372 (Δ*4* Δ*ltaS* Δ*yqgS*), AG377 (Δ*4* Δ*yfnI* Δ*yqgS*), and AG380 (Δ*4* Δ*ltaS* Δ*yfnI* Δ*yqgS*). Samples were inoculated onto the same NA plates with or without 1% glucose and incubated at 37°C for 24 h. * indicates that the Δ*4* Δ*yfnI* strain was found to pick up suppressor mutations more rapidly than AG342. (C) Tenfold serial dilutions were prepared for B. subtilis strains 168CA (wild type), AG223 (Δ*4* Δ*mprF*), AG389 (AG223 Δ*ltaS* Δ*yqgS*), AG390 (AG223 Δ*yfnI* Δ*yqgS*), AG399 (AG223 Δ*ltaS* Δ*yfnI*), and AG400 (AG223 Δ*yqgS* Δ*ltaS* Δ*yfnI*) and spotted on the same plates. Plates (with 1% glucose here) were incubated at 37°C and imaged after 24 h. (D) Representative microscopy images of cells of strains from panel B grown in NB at 37°C for 120 min and stained with FM5-95 dye. Bar, 5 μm. The average cell diameters from Table 4 are indicated. * indicates that the cell measurement was done on the straight regions of the cell chain. Strain phenotypes were tested at least three times. Supporting information is presented in Fig. S2C and Fig. S3 in the supplemental material, which also display results obtained in the Δ*ponA* background.

To comprehend the effect of the loss of MprF on a strain lacking aPBPs and identify the MprF suppression pathway, we carried out a transposon screen in a strain lacking the vegetative class A PBPs, *mprF*, and *lacA* and carrying pLOSS-*P_spac_*-*ponA* (strain AG200) (Tables 1 and 2) (12), with the objective of identifying alleles that rendered the maintenance of the plasmid copy of *ponA* essential. From this screen, strain AG200BK#42 was isolated with the expected phenotype and was found to carry a transposon inserted into the *gtaB* gene. GtaB catalyzes the cytoplasmic conversion of glucose-1-phosphate (Glc-1-P) to UDP-Glc (Fig. 1), where the latter ultimately contributes to LTA synthesis and WTA modification. The introduction of a *gtaB* deletion into the Δ*4* and Δ*4* Δ*mprF* backgrounds resulted in lethal (NA-glucose) and sick (NA) phenotypes (Fig. 3A), confirming the conditional essentiality of GtaB.

10.1128/mbio.02667-22.3 FIG S3 Cell morphology changes associated with deletions of LTA synthase genes combined with class A PBP-null mutants of B. subtilis. (A and B) All of the strains grew exponentially in NB with 10 mM MgSO_4_ and were washed and diluted in NB. Strains 168CA, AG157 (Δ*4*), AG342 (Δ*4* Δ*ltaS*), AG377 (Δ*4* Δ*yfnI* Δ*yqgS*), AG370 (Δ*4* Δ*ltaS* Δ*yfnI*), AG372 (Δ*4* Δ*ltaS* Δ*yqgS*), AG380 (Δ*4* Δ*ltaS* Δ*yfnI* Δ*yqgS*), and AG223 (Δ*4* Δ*mprF*) (Table 2) were grown for 120 min until mid-exponential phase at 37°C. Cells were stained with FM5-95 dye, and microscopy images were acquired. Cells were measured using the ObjectJ plug-in in ImageJ. About 400 cells were counted for each strain. The results shown here are from data acquired from one experiment only. Due to the formation of twisted cell chains, the cell measurement of the Δ*4* Δ*ltaS* Δ*yfnI* Δ*yqgS* strain was done on the straight regions of the chain. Distributions of the cell width (A) and cell length (B) are shown. Table 4 indicates the average cell widths and cell lengths of the cell populations analyzed. (C and D) Effects of LTA synthase deletions in the Δ*ponA* background. (C) Tenfold serial dilutions were prepared in NB as described above for strains 168CA, RE101 (Δ*ponA*), AG383 (Δ*ponA* Δ*ltaS*), AG384 (Δ*ponA* Δ*yfnI*), AG385 (Δ*ponA* Δ*yqgS*), AG393 (Δ*ponA* Δ*ltaS* Δ*yfnI*), AG394 (Δ*ponA* Δ*ltaS* Δ*yqgS*), AG395 (Δ*ponA* Δ*yfnI* Δ*yqgS*), and AG403 (Δ*ponA* Δ*ltaS* Δ*yfnI* Δ*yqgS*) (Table 2). Plates were incubated at 37°C and imaged at 22 h. (D) The strains described above for panel C were diluted in NB and grown at 37°C for 120 min. When cells reached mid-exponential phase, cell membranes were stained with FM5-95 dye and observed under a microscope. Representative images were assembled using Photoshop. Bar, 5 μm. Download FIG S3, TIF file, 1.6 MB. Copyright © 2023 Guyet et al. 2023 Guyet et al. https://creativecommons.org/licenses/by/4.0/ This content is distributed under the terms of the Creative Commons Attribution 4.0 International license.

To determine if this was related to LTA synthesis or WTA modification, we deleted sequentially, in the Δ*4* or Δ*4*/pLOSS-*P_spac_*-*ponA* background, each enzyme whose function is immediately upstream or downstream of GtaB (Fig. 1B). Characterization of the resulting strains (Table 2) showed that only the deletion of *ugtP*, encoding the enzyme that produces the Glc_2_DAG lipid anchor for LTA, had an effect similar to that of *gtaB*. The Δ*4* Δ*mprF* Δ*ugtP* mutant was unable to grow on NA-glucose (Fig. 3A), a phenotype common to Δ*ponA* Δ*gtaB* and Δ*ponA* Δ*ugtP* mutants (Fig. S2C). On NA, the Δ*4* Δ*gtaB* and Δ*4* Δ*ugtP* mutants grew very slowly (Fig. 3A, right).

The level of LysPGol is known to increase in Δ*ugtP* cells (24), which could explain the similarity of the phenotypes of the Δ*4* Δ*ugtP* and Δ*4* MprF↑ mutants (i.e., slow growth on NA) but not the dominant effect of the Δ*ugtP* mutation on the Δ*4* Δ*mprF* mutant. In S. aureus, it has been observed that Δ*ugtP* (*ypfP*) and Δ*gtaB* mutants produce long LTAs (32). Those and our results together reinforced the idea that MprF may have a role in LTA synthesis.

### The deletion of *ltaS* improves the conditional lethality of a strain lacking aPBPs.

To verify that LTA synthesis alters Δ*4* growth, we analyzed the phenotypes of strains lacking aPBPs and the LTA synthase genes individually or in combination (Fig. 1). Surprisingly, the Δ*4* Δ*ltaS* mutant was viable on NA-glucose, even when *yfnI* or *yqgS* was also deleted (Fig. 3B). Conversely, strains expressing LtaS showed limited growth and were found to pick up suppressor mutations rapidly on NA-glucose (e.g., Δ*4* Δ*yfnI* and Δ*4* Δ*yfnI* Δ*yqgS*) (Fig. 3B). Thus, LtaS activity also contributes to the glucose-associated lethality of the Δ*4* mutant. Interestingly, the absence of MprF is more advantageous to the Δ*4* mutant than the absence of LtaS, as it results in faster growth and larger colonies on NA-glucose (Fig. S1B). This phenotypic difference indicated that slightly different mechanisms helped restore the growth of the Δ*4* mutant.

To test this, the LTA synthase genes were systematically deleted in the Δ*4* Δ*mprF* (AG223) background, and the viability of the mutants generated was determined on NA with or without glucose. All of the strains remained viable on NA-glucose (Fig. 3C), suggesting that the effect of the loss of MprF could be related to LTA. In addition, in the Δ*4* Δ*mprF* background, the combined absence of *ltaS* and *yfnI* delayed growth on NA-glucose, resulting in a phenotype similar to that observed on NA (Fig. 3C). Thus, in these specific genetic backgrounds, LTA synthesis is not essential but permits higher growth efficiencies.

The absence of neither LtaS nor MprF had any effect on the filamentous thin-cell morphology of the Δ*4* mutant (Table 4 and Fig. S3A and B). In the absence of all three LTA synthases, B. subtilis is viable, but the cells are filamentous and clumpy (16). We also observed that the combined absence of LtaS, YfnI, and/or YqgS in the Δ*4*, Δ*ponA*, or Δ*4* Δ*mprF* genetic background led to an increase in the cell diameter, although cells remained linked at the site of division (Fig. 3D, Table 4, and Fig. S3A and B). However, when LtaS was the only functional LTA synthase (e.g., Δ*4* Δ*yqgS* Δ*yfnI*) (Fig. 3D), cells were thin and comparable to those of the Δ*4* and Δ*ponA* mutants (Fig. S3C and D). Collectively, our results indicate that the activity of the LTA synthases, particularly LtaS, could account for the Δ*4* phenotype.

**TABLE 4:** Cell morphogenesis changes associated with deletions of LTA synthase genes combined with class A PBP-null mutants of B. subtilis^*a*^

Strain	Cell width (μm)		Cell length (μm)		No. of cells counted
	Avg	SD	Avg	SD	
168CA	0.8399	0.0469	3.258	0.787	437
Δ*4*	0.6595	0.0492	4.557	2.075	419
Δ*4* Δ*mprF*	0.6793	0.0546	4.659	2.18	399
Δ*4* Δ*ltaS*	0.6679	0.055	4.828	2.325	414
Δ*4* Δ*ltaS* Δ*yfnI*	0.7269	0.0528	4.873	2.24	426
Δ*4* Δ*ltaS* Δ*yqgS*	0.756	0.0499	4.926	2.301	420
Δ*4* Δ*yfnI* Δ*yqgS*	0.6538	0.0513	4.813	1.926	423
Δ*4* Δ*ltaS* Δ*yfnI* Δ*yqgS*^*b*^	0.7666	0.0529	5.312	2.651	416

a The average cell widths and cell lengths of strains 168CA, AG157 (Δ*4*), AG342 (Δ*4* Δ*ltaS*), AG377 (Δ*4* Δ*yfnI* Δ*yqgS*), AG370 (Δ*4* Δ*ltaS* Δ*yfnI*), AG372 (Δ*4* Δ*ltaS* Δ*yqgS*), AG380 (Δ*4* Δ*ltaS* Δ*yfnI* Δ*yqgS*), and AG223 (Δ*4* Δ*mprF*) are analyzed in Fig. S3A and B in the supplemental material. Standard deviations are indicated.

b Due to the formation of twisted cell chains, the cell measurement of the Δ*4* Δ*ltaS* Δ*yfnI* Δ*yqgS* strain was done on the straight regions of the chain.

### Impact of glucose, PBP1, and MprF on LTA synthesis.

**FIG 4:**
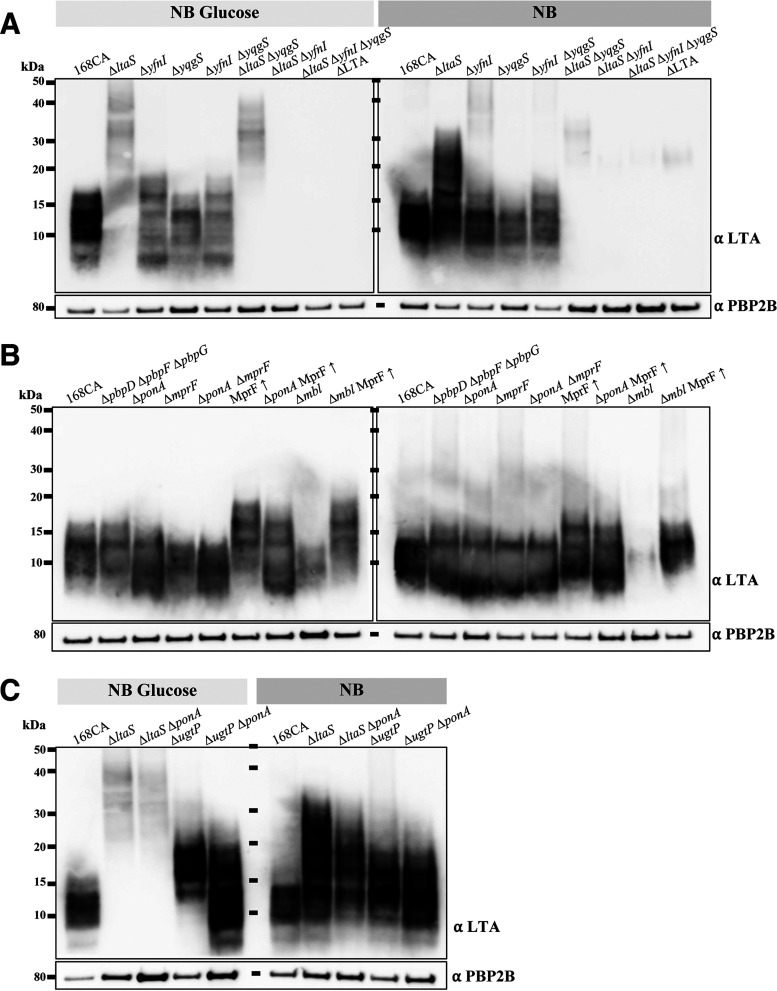
Analysis of lipoteichoic acid production in B. subtilis strains. For each set of cultures (NB with or without 0.2% glucose), samples were separated on the same Bis-Tris gradient gel. The membrane was split at the ~60-kDa position. The lower part of the membrane was probed using a monoclonal antibody for Gram-positive LTA and an HRP-linked anti-mouse antibody. The other membrane part was probed using polyclonal anti-PBP2B and HRP-linked anti-rabbit antibodies to provide a sample loading control. (A) Samples were prepared from B. subtilis strains 168CA (wild type), 4285CA (Δ*ltaS*), 4289CA (Δ*yfnI*), 4292CA (Δ*yqgS*), AG595 (Δ*yfnI* Δ*yqgS*), AG593 (Δ*ltaS* Δ*yqgS*), AG594 (Δ*ltaS* Δ*yfnI*), AG600 (Δ*ltaS* Δ*yfnI* Δ*yqgS*), and 4620 (Δ*ltaS* Δ*yfnI* Δ*yqgS* Δ*yvgJ* [denoted ΔLTA here]). (B) Analysis of B. subtilis strains 168CA, AG417 (Δ*pbpD* Δ*pbpF* Δ*pbpG*), RE101 (Δ*ponA*), AG181 (Δ*mprF*), AG193 (Δ*ponA* Δ*mprF*), AG304 (MprF↑), AG311 (Δ*ponA* MprF↑), 4261CA (Δ*mbl*), and AG322 (Δ*mbl* MprF↑). A densitometry graph of the left blot is presented in Fig. S5D in the supplemental material. (C) Samples were extracted from B. subtilis strains 168CA, 4285CA (Δ*ltaS*), AG383 (Δ*ltaS* Δ*ponA*), PG253 (Δ*ugtP*), and AG444 (Δ*ugtP* Δ*ponA*). The results are representative of data from one of three independent experiments shown in Fig. S4 to Fig. S6. In each section of panels A to C, samples were derived from the same experiment.

To comprehend the effects of LtaS, MprF, and UgtP in the absence of aPBPs, we decided to analyze LTA production. We developed a Western blot protocol relying on a monoclonal antibody for Gram-positive LTA (Thermo Fisher) and a polyclonal PBP2B antibody (as a control). First, we determined the LTA profiles for B. subtilis wild-type (WT) strain 168CA and the LTA synthase mutants (Fig. 4A and Fig. S4) grown in NB and NB-glucose (0.2%). The wild-type LTA signal corresponded to a range of mobilities between 7 and 17 kDa, as previously identified (18). Interestingly, the LTA signal in Δ*ltaS* cells grown in NB-glucose (>15 kDa) (Fig. 4A, left, 2nd lane) was distinct from that seen for growth in NB (7 to 32 kDa) (Fig. 4A, right, 2nd lane). However, the LTA pattern was different when YfnI was the only functional LTA synthase (Fig. 4A). This analysis also revealed that short LTAs (<10 kDa) were more abundantly produced in NB-glucose. This seemed to be related to LtaS activity, as we observed an increase in short LTAs in the Δ*yfnI* and Δ*yfnI* Δ*yqgS* mutants compared to 168CA (Fig. 4A, left, 3rd and 5th lanes). Collectively, our results show that the regulation of LTA synthesis is more complex than previously thought (18) as it is influenced by the metabolic/nutritional status of the cells.

10.1128/mbio.02667-22.4 FIG S4 Results of three independent LTA Western blot assays supporting the data in Fig. 4A. The lower part of the membrane was probed using a monoclonal LTA antibody and an HRP-linked anti-mouse antibody. The other membrane part was probed using polyclonal anti-PBP2B and HRP-linked anti-rabbit antibodies to provide a sample loading control. Samples were prepared from B. subtilis strains 168CA (wild type), 4285CA (Δ*ltaS*), 4289CA (Δ*yfnI*), 4292CA (Δ*yqgS*), AG595 (Δ*yfnI* Δ*yqgS*), AG593 (Δ*ltaS* Δ*yqgS*), AG594 (Δ*ltaS* Δ*yfnI*), AG600 (Δ*ltaS* Δ*yfnI* Δ*yqgS*), and 4620 (Δ*ltaS* Δ*yfnI* Δ*yqgS* Δ*yvgJ* [denoted ΔLTA here]). Download FIG S4, TIF file, 1.3 MB. Copyright © 2023 Guyet et al. 2023 Guyet et al. https://creativecommons.org/licenses/by/4.0/ This content is distributed under the terms of the Creative Commons Attribution 4.0 International license.

10.1128/mbio.02667-22.5 FIG S5 Results of three independent LTA Western blot assays supporting the data in Fig. 4B. (A to C) The lower part of the membrane was probed using a monoclonal LTA antibody and an HRP-linked anti-mouse antibody. The other membrane part was probed using polyclonal anti-PBP2B and HRP-linked anti-rabbit antibodies to provide a sample loading control. Samples were prepared from B. subtilis strains 168CA (wild type), AG417 (Δ*pbpD* Δ*pbpF* Δ*pbpG*), RE101 (Δ*ponA*), AG181 (Δ*mprF*), AG193 (Δ*ponA* Δ*mprF*), AG304 (MprF↑), AG311 (Δ*ponA* MprF↑), 4261CA (Δ*mbl*), and AG322 (Δ*mbl* MprF↑). (D) The LTA signals of some of the strains grown with glucose are represented as densitometry graphs below their corresponding LTA blots. Analysis was done for each biological replicate presented in either Fig. 4B (panel B) or panels A and C in this figure. Raw blot images were analyzed using ImageJ software and the “analysis gels” function after a same-sized area was defined for each lane. The dashed lines provide guidelines to help compare the shifts of LTA lengths between samples relative to the wild type. Download FIG S5, TIF file, 13.1 MB. Copyright © 2023 Guyet et al. 2023 Guyet et al. https://creativecommons.org/licenses/by/4.0/ This content is distributed under the terms of the Creative Commons Attribution 4.0 International license.

10.1128/mbio.02667-22.6 FIG S6 Results of three independent LTA Western blot assays supporting the data in Fig. 4C. The lower part of the membrane was probed using a monoclonal LTA antibody and an HRP-linked anti-mouse antibody. The other membrane part was probed using polyclonal anti-PBP2B and HRP-linked anti-rabbit antibodies to provide a sample loading control. Samples were extracted from B. subtilis strains 168CA, 4285CA (Δ*ltaS*), AG383 (Δ*ltaS* Δ*ponA*), PG253 (Δ*ugtP*), and AG444 (Δ*ugtP* Δ*ponA*). Download FIG S6, TIF file, 0.9 MB. Copyright © 2023 Guyet et al. 2023 Guyet et al. https://creativecommons.org/licenses/by/4.0/ This content is distributed under the terms of the Creative Commons Attribution 4.0 International license.

If the *mprF* mutation suppressed the Δ*ponA* phenotype by altering LTA synthesis (Fig. 2A and Fig. S1B), a change in the LTA profile might be detectable. To test this, we used the Δ*ponA* mutant, where only the major aPBP was deleted, as the multiple-aPBP-deletion strain was very lytic and did not grow under certain conditions (Fig. S1). Western blot analysis of Δ*ponA* cells grown in the presence of glucose at 0.2% (Fig. 4B, left, 3rd lane) revealed a shift toward shorter LTAs than those of the wild type (Fig. S5D). This increase in short LTAs was not evident in the absence of glucose. Interestingly, the LTA signal of the Δ*ponA* Δ*mprF* mutant decreased compared to that of the Δ*ponA* mutant (Fig. 4B, left, 5th lane), and the Δ*mprF* mutant (Fig. 4B, left, 4th lane) showed a decrease in the LTA signal compared to that of 168CA in NB-glucose (Fig. S5D). In the absence of *mprF*, both strains had a reduction in the LTA length (Fig. S5D). We next examined the effects of *mprF* overexpression (MprF↑) (Fig. 4B, 6th lane), and we were surprised to see increases in the LTA polymer length and range (Fig. S5D). This effect was dependent on LtaS but not YfnI, as a similar LTA profile was observed for the Δ*yfnI* MprF↑ strain, while the profile of the Δ*ltaS* MprF↑ strain was similar to that of the Δ*ltaS* strain (strains AG353 and AG349 in Table 2) (data not shown). The latter was expected as LtaS activity seems dominant over YfnI activity (18). The overexpression of *mprF* in Δ*ponA* cells resulted in a broader size range of LTAs than that seen for the wild type and the Δ*ponA* mutant, as we observed an increase in the LTA signals below 10 kDa but also above 15 kDa (Fig. 4B, and Fig. S5D 7th lanes). To some extent, this resembled what was observed in the Δ*ponA* and MprF↑ single mutants except that the LTA polymers of the Δ*ponA* MprF↑ mutant were not as long as those of the MprF↑ single mutant (Fig. S5D). Thus, the cumulative effect of the mutations led to an increase in the LTA length range and could explain the lethality of the Δ*ponA* MprF↑ double mutant on NA-glucose-IPTG (isopropyl-β-d-thiogalactopyranoside) (Fig. 2B).

In contrast to the Δ*ponA* mutant, the overexpression of *mprF* in the Δ*mbl* mutant rescued its growth (Fig. 2B). To see whether this viability was associated with a change in the LTA profile, we analyzed the two strains by Western blotting. The LTA size range of the Δ*mbl* mutant was shifted toward shorter LTAs, and the overall LTA signal was weaker than that of the wild type (Fig. 4B, and Fig. S5D second to last lanes). The overexpression of *mprF* in the Δ*mbl* mutant resulted in increases in both the LTA length and abundance (Fig. 4B, last lane). Longer LTA polymers, similar to those of the Δ*ltaS* mutant (Fig. 4A), were also observed in the Δ*mbl* Δ*ltaS* mutant (data not shown). Thus, an increase in the LTA length seems to characterize the effects of both Δ*mbl* suppressor mutations, suggesting that the presence of short and/or less abundant LTAs could be a factor contributing to the growth defect of the Δ*mbl* mutant.

Analysis of the Δ*ltaS* Δ*ponA* mutant revealed the dominance of the *ltaS* deletion in that the LTAs exhibited retarded migration (Fig. 4C and Fig. S6, 3rd lanes) similar to that observed for the Δ*ltaS* mutant (Fig. 4C). In the absence of UgtP, which produces a lipid anchor, Glc_2_DAG (Fig. 1), we observed an increase in the LTA length in both media, but the shift was more distinct with glucose (>12 kDa) (Fig. 4C, left, 4th lane). The latter is comparable to the LTA signal detected previously by Gründling and Schneewind (32) for the S. aureus Δ*ugtP* mutant, which showed an LTA size range higher than that of the wild type when grown in tryptic soy broth (TSB), a glucose-rich medium. Analysis of the Δ*ponA* Δ*ugtP* mutant, which was conditionally lethal on NA-glucose (Fig. S2C), revealed increases in both the LTA signal and length (7 to 25 kDa) (Fig. 4C, left, 5th lane), a pattern reminiscent of that determined for the Δ*ponA* MprF↑ strain (Fig. 4B, left, 7th lane), which was also lethal on NA-glucose (Fig. 2B).

To conclude, we show for the first time that MprF has a role in altering the length of LTAs through an LtaS-dependent mechanism and has an impact on cell wall metabolism. Our results also provide strong evidence that the conditions leading to the lethality of *ponA* are associated with increases in the abundance and length of LTAs.

### Overexpression of the major autolysin LytE is lethal in the absence of PBP1 or LtaS.

Our results implied that the conditional lethality of the aPBP mutants might be linked to an interplay between the LTA length and autolysin activity. Autolysins or cell wall hydrolases help shape and recycle the peptidoglycan during cell elongation and division. B. subtilis expresses a large number of them, but only the loss of both of the PG d,l-endopeptidases CwlO and LytE is lethal (13, 14, 44–46) (Fig. 1A). We focused on LytE because it is considered to be one of the key autolysins, and it is secreted and thought to be anchored to the lateral cell wall and septum in a way that is influenced by teichoic acids (43). The abundance of LytE is also increased in a Δ*ltaS* mutant (47), and both LtaS and LytE affect colony development (48) and cell diameter (16, 43).

**FIG 5:**
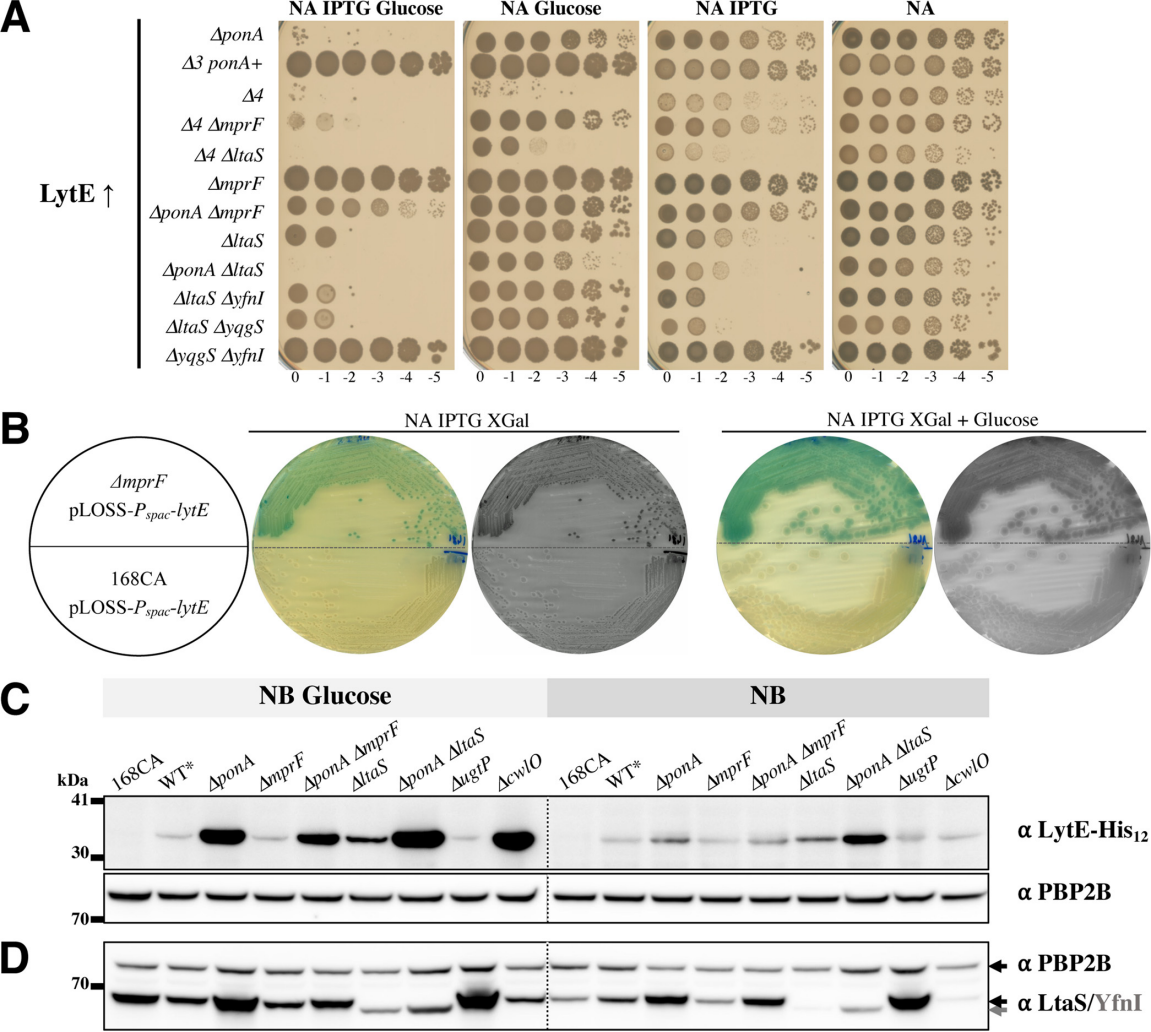
LytE overexpression is conditionally lethal in the absence of PBP1 or LtaS. (A) *lytE* overexpression was analyzed in strains that integrated IPTG-inducible *P_hyspank_-lytE* at the *amyE* locus (in the presence of 0.1 mM IPTG), represented as LytE↑ here. The following tested strains with relevant features are shown: AG484 (Δ*ponA* LytE↑), AG1460 (Δ*3 ponA*^+^ LytE↑), AG501 (Δ*4* LytE↑), AG502 (Δ*4* Δ*mprF* LytE↑), AG1465 (Δ*4* Δ*ltaS* LytE↑), AG1478 (Δ*mprF* LytE↑), AG1479 (Δ*ponA* Δ*mprF* LytE↑), AG1462 (Δ*ltaS* LytE↑), AG1480 (Δ*ponA* Δ*ltaS* LytE↑), AG1497 (Δ*ltaS* Δ*yfnI* LytE↑), AG1498 (Δ*ltaS* Δ*yqgS* LytE↑), and AG1499 (Δ*yqgS* Δ*yfnI* LytE↑). The following strains that did not display any obvious phenotype are not represented here: AG475 (168CA LytE↑), AG1461 (Δ*lytE* LytE↑), AG1463 (Δ*yfnI* LytE↑), and AG1492 (Δ*yqgS* LytE↑). Plates (with 0.5% glucose here) were incubated at 37°C and scanned after 24 h (this figure) and 48 h (see Fig. S7A in the supplemental material). (B) Strains AG1481 (Δ*mprF* pLOSS-*P_spac_-lytE*) and AG551 (168CA carrying pLOSS-*P_spac_-lytE*) were streaked onto NA and NA-glucose (0.5%) supplemented with IPTG (1 mM) and X-Gal from a fresh colony on NA-spectinomycin. Plates were incubated at 37°C and scanned after 24 h. Blue colonies indicate the stabilization of plasmid pLOSS-*P_spac_-lytE* in the Δ*mprF* strain. Grayscale images are shown here to help visualize white colonies. (C) Detection of LytE-His_12_ and PBP2B by Western blotting. For simplification, only the relevant background features are displayed for the following strains (expressing LytE-His_12_ under the control of its native promoter): wild-type-like AG565 (WT*), AG587 (Δ*ponA*), AG1486 (Δ*mprF*), AG1487 (Δ*ponA* Δ*mprF*), AG1489 (Δ*ltaS*), AG1488 (Δ*ponA* Δ*ltaS*), AG1535 (Δ*ugtP*), and AG1541 (Δ*cwlO*). In addition, B. subtilis 168CA was grown in parallel (without antibiotics) and used here as a negative control for the LytE blot. The membrane was split and incubated with either monoclonal penta-His and HRP-linked anti-mouse antibodies or polyclonal anti-PBP2B and HRP-linked anti-rabbit antibodies. Note that a “ladder” sample loaded after the NB-glucose (0.2%) sample set is not displayed, and the image editing is symbolized by the vertical black line (see the original in Fig. S7B, middle). (D) Detection of the LTA synthases LtaS/YfnI and PBP2B by Western blotting. The samples used in the experiment in panel C were loaded onto a new SDS gel, and Western blotting was carried out with polyclonal anti-LtaS (able to detect YfnI [J. Errington, unpublished data]), polyclonal anti-PBP2B, and HRP-linked anti-rabbit antibodies. The results from one representative set of three independent Western blot experiments are shown here and in Fig. S7B and Fig. S8A. Additional supporting information is presented in Fig. S8 and Fig. S9. In each section, samples were derived from the same experiment.

We reasoned that if an imbalance of LytE activity existed in the aPBP mutants (Δ*4* and Δ*ponA*) grown in glucose-rich medium, then a deliberate increase in LytE expression should exacerbate the strain phenotype, leading to cell death. Interestingly, *lytE* overexpression (by IPTG induction of a *P_hyspank_-lytE* allele) was lethal in both the Δ*4* and Δ*ponA* backgrounds when cells were grown on NA-glucose (Fig. 5A and Fig. S7A), and it significantly delayed their growth on NA but not that of the Δ*3 ponA*^+^ strain. This result confirmed that an imbalance in autolytic activity was detrimental to the cells that had lost PBP1. Importantly, the overexpression of *lytE* in the Δ*4* Δ*ltaS*, Δ*ponA* Δ*ltaS*, and Δ*ltaS* strains was also lethal (Fig. 5A), while the growth of the Δ*ponA* Δ*mprF* strain was only slightly delayed. The Δ*mprF* single mutant was also unaffected by *lytE* overexpression. In contrast, the Δ*4* Δ*mprF* mutant was viable when *lytE* was overexpressed, although its growth was severely perturbed on glucose.

10.1128/mbio.02667-22.7 FIG S7 Abundance of LytE in the cell envelope and growth characteristics of the strains. (A) LytE overexpression is conditionally lethal in the absence of PBP1 or LtaS. *lytE* overexpression was analyzed in strains that integrated IPTG-inducible *P_hyspank_-lytE* at the *amyE* locus, represented here as LytE↑. The following strains with relevant features were tested: AG484 (Δ*ponA* LytE↑), AG1460 (Δ*3 ponA^+^* LytE↑), AG501 (Δ*4* LytE↑), AG502 (Δ*4* Δ*mprF* LytE↑), AG1465 (Δ*4* Δ*ltaS* LytE↑), AG1478 (Δ*mprF* LytE↑), AG1479 (Δ*ponA* Δ*mprF* LytE↑), AG1462 (Δ*ltaS* LytE↑), AG1480 (Δ*ponA* Δ*ltaS* LytE↑), AG1497 (Δ*ltaS* Δ*yfnI* LytE↑), AG1498 (Δ*ltaS* Δ*yqgS* LytE↑), and AG1499 (Δ*yqgS* Δ*yfnI* LytE↑). The following strains that did not display any obvious phenotype are not represented here: AG475 (168CA LytE↑), AG1461 (Δ*lytE* LytE↑), AG1463 (Δ*yfnI* LytE↑), and AG1492 (Δ*yqgS* LytE↑). NA plates were supplemented with IPTG (0.1 mM), glucose (Glc) (0.5%), and MgSO_4_ (10 mM). Plates were incubated at 37°C and scanned after 24 h (Fig. 5A) and 48 h. (B) Results of three independent experiments supporting the data in Fig. 5C. The relative abundances of the LytE and PBP2B proteins in mutant strains were detected by Western blotting. Strains expressing LytE-His_12_ under the control of its native promoter were grown in NB and NB supplemented with 0.2% glucose until the late exponential growth phase at 37°C. For simplification, only the following strains with relevant background features are displayed: wild-type-like AG565 (WT*), AG587 (Δ*ponA*), AG1486 (Δ*mprF*), AG1487 (Δ*ponA* Δ*mprF*), AG1489 (Δ*ltaS*), AG1488 (Δ*ponA* Δ*ltaS*), AG1535 (Δ*ugtP*), and AG1541 (Δ*cwlO*). In addition, wild-type strain 168CA (i.e., not expressing LytE-His_12_) was grown in parallel and used here as a negative control for the LytE blot. The bottom part of the membrane (split at the ~53-kDa position) was incubated with monoclonal penta-His and HRP-linked anti-mouse antibodies. The top membrane part was detected with polyclonal anti-PBP2B and HRP-linked anti-rabbit antibodies. #For each section, the middle panel corresponds to the raw image of the LytE-His_12_ blot at a detection time where one of the sample signals had reached saturation. Download FIG S7, TIF file, 2.1 MB. Copyright © 2023 Guyet et al. 2023 Guyet et al. https://creativecommons.org/licenses/by/4.0/ This content is distributed under the terms of the Creative Commons Attribution 4.0 International license.

10.1128/mbio.02667-22.8 FIG S8 Data supporting the results in Fig. 5. (A) Results of three independent experiments supporting the data in Fig. 5D. The relative abundances of the LTA synthases LtaS, YfnI, and PBP2B in mutant strains were determined by Western blotting. The samples used in Fig. S7B were loaded onto a new SDS gel, and the Western blot was developed with polyclonal anti-LtaS (which cross-reacts with YfnI [Errington, unpublished]), polyclonal anti-PBP2B, and HRP-linked anti-rabbit antibodies. A weak nonspecific protein band was observed between PBP2B and LtaS and was detected by the anti-PBP2B antibody. Strains expressing LytE-His_12_ under the control of its native promoter were grown in NB and NB supplemented with glucose (0.2%) until late exponential phase at 37°C. For simplification, only the following strains with relevant background features are displayed: 168CA, wild-type-like AG565 (WT*), AG587 (Δ*ponA*), AG1486 (Δ*mprF*), AG1487 (Δ*ponA* Δ*mprF*), AG1489 (Δ*ltaS*), AG1488 (Δ*ponA* Δ*ltaS*), AG1535 (Δ*ugtP*), and AG1541 (Δ*cwlO*). (B) Accumulation of LtaS-His and YfnI-His in Δ*ponA* cells. Strains were grown in NB and NB supplemented with 0.2% glucose until the late exponential growth phase at 37°C. The strains were tested once under conditions similar to those for the protein Western blots presented in this study, following a preliminary test. *The strains used here express the following His-tagged proteins under the control of their native promoters: LtaS-His_12_ in a wild-type-like (AG569) or a Δ*ponA* (AG588) background and YfnI-His_12_ in a wild-type-like (WT) (AG570 strain) or a Δ*ponA* (AG589) background. His-tagged proteins were detected with monoclonal penta-His and HRP-linked anti-mouse antibodies. The membrane was reused to detect PBP2B (sample loading control) with polyclonal anti-PBP2B and HRP-linked anti-rabbit antibodies (second panel). The experiment was performed on one biological set of samples. (C) Accumulation of LtaS/YfnI and LytE in LTA synthase mutants. The following strains were grown (NB with or without 0.2% glucose) under conditions similar to those of our previous assays: B. subtilis 168CA, 4285CA (Δ*ltaS*), 4289CA (Δ*yfnI*), 4292CA (Δ*yqgS*), AG595 (Δ*yqgS* Δ*yfnI*), AG593 (Δ*ltaS* Δ*yqgS*), AG594 (Δ*ltaS* Δ*yfnI*), and AG600 (Δ*ltaS* Δ*yfnI* Δ*yqgS*). Here, the Western blot experiments were carried out under the same conditions as those of the other assays (polyclonal anti-LtaS antibody cross-reacts with YfnI [Errington, unpublished]) (A), except for the use of our newly produced polyclonal anti-LytE antibody (C). The experiment was performed on one biological set of samples. Download FIG S8, TIF file, 1.0 MB. Copyright © 2023 Guyet et al. 2023 Guyet et al. https://creativecommons.org/licenses/by/4.0/ This content is distributed under the terms of the Creative Commons Attribution 4.0 International license.

10.1128/mbio.02667-22.9 FIG S9 Effect of magnesium in culture medium on the production of LytE and LtaS and the accumulation of LtaS/YfnI and LytE in the synthetically sick Δ*ponA* MprF↑ and Δ*ponA* Δ*ugtP* strains. Detection of LytE-His_12_ and PBP2B was performed by Western blotting. Strains expressing LytE-His_12_ under the control of its native promoter were grown in NB supplemented with 0.2% glucose with or without MgSO_4_ (10 mM) at 37°C. For simplification, here, only the following strains with the relevant background features are displayed: wild-type-like AG565 (WT*), AG587 (Δ*ponA*), AG1487 (Δ*ponA* Δ*mprF*), AG1488 (Δ*ponA* Δ*ltaS*), AG1541 (Δ*cwlO*), AG1684 (Δ*ponA* MprF↑), and AG1685 (Δ*ponA* Δ*ugtP*). The last two strains were also grown in parallel in NB. The top membrane part was incubated with polyclonal anti-LtaS (which cross-reacts with YfnI [Errington, unpublished]), polyclonal anti-PBP2B, and HRP-linked anti-rabbit antibodies. The bottom part of the membrane was incubated with monoclonal penta-His and HRP-linked anti-mouse antibodies. Images were processed using ImageJ software. The results of three independent experiments are presented here. Download FIG S9, TIF file, 0.6 MB. Copyright © 2023 Guyet et al. 2023 Guyet et al. https://creativecommons.org/licenses/by/4.0/ This content is distributed under the terms of the Creative Commons Attribution 4.0 International license.

These observations suggested that the Δ*ponA* Δ*ltaS* and Δ*ponA* Δ*mprF* double mutants, which have different LTA length ranges, were differently sensitive to LytE activity. Therefore, we examined the LTA synthase double mutants to see how they reacted to an increase in *lytE* expression. We found that when *lytE* was overexpressed, the LTA synthase double mutants were viable only if *ltaS* was still present (Fig. 5A, bottom strain), and the deletion of *mprF* or *ugtP* from these mutants had no effect on strain viability (Table 2 and data not shown). Interestingly, the Δ*ltaS* LytE↑ mutant was viable on NA (Fig. 5A, 8th strain) but not on NA-glucose, which is probably related to the above-described observation that the Δ*ltaS* mutant produced LTAs of different lengths on these two media (Fig. 4A). Thus, it is clear that the activity of LtaS is required for the cell to balance the effect of *lytE* overexpression, presumably by producing LTAs of a specific length.

To comprehend the apparent tolerance of LtaS^+^ and Δ*mprF* cells to LytE overexpression, we constructed an unstable replicon plasmid, pLOSS-*P_spac_*-*lytE*, which expresses *lytE* at a lower level than *P_hyspank_-lytE* (Table 1). This plasmid will be retained by the cell only if *lytE* expression benefits the cells. In the Δ*mprF* mutant (AG1481 strain), the pLOSS-*P_spac_*-*lytE* plasmid was retained under all medium conditions tested, including elevated magnesium levels, as indicated by the blue color of the colonies on plates supplemented with IPTG–X-Gal (5-bromo-4-chloro-3-indolyl-β-d-galactopyranoside) (Fig. 5B). However, in the other strains tested (e.g., 168CA, Δ*ponA*, Δ*ponA* Δ*mprF*, and LTA synthase mutants) (Table 2), the plasmid was frequently lost, an indication that there was no selective advantage for the cells to maintain the plasmid. At this stage, it is unclear if the Δ*mprF* strain’s tolerance of *lytE* overexpression is due to the loss of aaPGols, the production of shorter LTAs, or both.

### LTA influences LytE activity.

The conditional lethality observed when *lytE* was overexpressed prompted us to investigate the cellular levels of this autolytic enzyme in our strains. First, we constructed strains that could express a recombinant protein, LytE-LEMGRSH_12_ (using pMUTin-′*lytE-his*) (Table 1), and then analyzed the total lysates by Western blotting using an antihistidine antibody (for LytE) (Fig. 5C and Fig. S7B) and an anti-LtaS antibody raised against LtaS (74 kDa) that cross-reacts with YfnI (73 kDa) (Fig. 5D and Fig. S8A). These analyses showed that LytE and LtaS/YfnI accumulated in Δ*ponA* cells, particularly when grown in the presence of glucose (Fig. 5C and D). Next, using a set of strains that carried either pMUTin-′*ltaS-his* or pMUTin-′*yfnI-his* integrated into the genome, we could confirm that both LtaS and YfnI were more abundant in Δ*ponA* cells (Tables 1 to 3 and Fig. S8B).

Importantly, in the Δ*ponA* Δ*mprF* strain, the LytE and LtaS/YfnI protein levels were lower than those in the Δ*ponA* strain (Fig. 5C and D), but no obvious change was observed in the Δ*mprF* mutant compared to the wild-type background expressing the LytE-LEMGRSH12 protein (denoted as WT*). On the contrary, analysis of the lytic Δ*ponA* MprF↑ and Δ*ponA* Δ*ugtP* strains cultured in either NB or NB-glucose showed that there were increased levels of LtaS/YfnI compared to those in the Δ*ponA* strain (Fig. S9B and C, 6 and 7th lanes). LytE was also more abundant in the Δ*ponA* Δ*ugtP* and Δ*ponA* MprF↑ mutants than in the wild type.

Finally, both the Δ*ltaS* and Δ*ponA* Δ*ltaS* mutants exhibited increases in the levels of both LytE and YfnI when grown in NB-glucose, an effect that was more pronounced for the Δ*ponA* Δ*ltaS* mutant (Fig. 5C and D, left). Therefore, in the absence of LtaS, the abundance of LytE increased. In addition, analysis of the set of LTA synthase mutants (Fig. S8C) revealed that the levels of LtaS/YfnI were increased in the presence of glucose; the level of LtaS was also increased significantly in the absence of YfnI. Therefore, our observations are consistent with the idea that the altered abundances of LytE and LtaS/YfnI proteins in Δ*ponA* cells were associated with the sick phenotype.

Taken together, our results show a correlation between the LTA length and the LytE abundance in the cell, which suggests that LTA acts to moderate cell wall metabolism through LytE. In *ponA* mutants, this function is unbalanced such that the activity of LytE, and, therefore, cell wall degradation, is predominant (summarized in Fig. 7).

### LTA production is altered in S. aureus Δ*mprF* and is an important factor in the sensitivity of B. subtilis to daptomycin.

**FIG 6:**
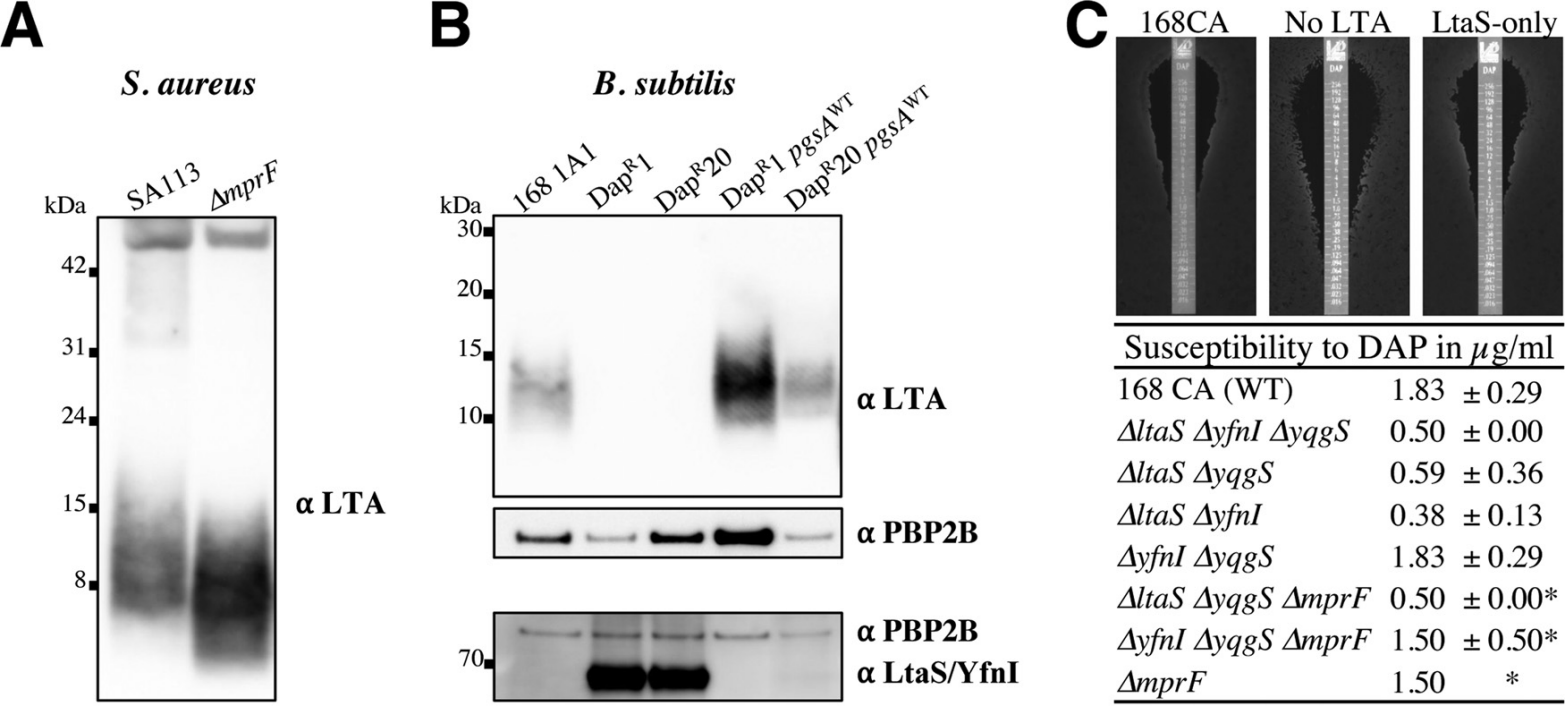
Daptomycin resistance and LTA synthesis in B. subtilis and S. aureus. (A) Samples of methicillin-sensitive Staphylococcus aureus strains SA113 and SA113 Δ*mprF* (21) were probed for LTA by Western blotting. (B) Researchers identified that a modified allele of *pgsA*, *pgsA^A64V^*, was the major determinant of the daptomycin resistance of the B. subtilis Dap^R^1 and Dap^R^20 strains selected by serial daptomycin antibiotic passages (54). (Top and middle) Strains 168 1A1 (wild type [WT]), Dap^R^1, Dap^R^20, HB15516 (Dap^R^1 *pgsA*^WT^), and HB15507 (Dap^R^20 *pgsA*^WT^) were grown in NB overnight at 30°C, and LTA was detected (top) along with PBP2B (middle) by Western blotting. Analysis of the samples grown exponentially at 37°C using these cultures grown overnight is presented in Fig. S10F in the supplemental material. (Bottom) Cellular abundance of the LTA synthase detected with an anti-LtaS antibody (which also cross-reacts with YfnI [Errington, unpublished]). Here, the samples were normalized based on the previous PBP2B blot (middle) (blotted as described in the legend of Fig. 5D). The results from one representative set of three experiments are shown here and in Fig. S10F. (C) The absence of the LTA synthase LtaS increases the susceptibility of B. subtilis to daptomycin. DAP strip assays (Liofilchem) on NA-CaCl_2_ plates were carried out on B. subtilis strains 168CA (wild type), AG600 (Δ*ltaS* Δ*yfnI* Δ*yqgS* [denoted No LTA]), AG593 (Δ*ltaS* Δ*yqgS*), AG594 (Δ*ltaS* Δ*yfnI*), AG595 (Δ*yfnI* Δ*yqgS* [denoted LtaS-only]), AG1673 (Δ*ltaS* Δ*yqgS* Δ*mprF*), AG1675 (Δ*yfnI* Δ*yqgS* Δ*mprF*), and AG1663 (Δ*mprF*). Plates were incubated at 37°C and scanned after 24 h (illustrations at the top). The table represents the averages from at least two independent experiments except for the Δ*mprF* strain (*). Additional supporting information is presented in Fig. S10.

MprF is conserved in the *Firmicutes* and has been shown to contribute to S. aureus pathogenicity (21, 22). Importantly, studies of clinical isolates have found that methicillin-resistant S. aureus (MRSA) strains that have acquired resistance to daptomycin, a lipopeptide antibiotic that acts to interfere with membranes containing phosphatidylglycerol (26, 49–51), frequently carry single nucleotide polymorphisms (SNPs) in the *mprF* gene (52, 53). Although some *mprF* SNPs were associated with an MprF gain of activity (increasing LysPGol and reducing PGol), others had no clear correlation between the phospholipid composition and the relative surface charge of the cell (26). In light of our results, we asked whether the newly discovered role of MprF in LTA production is conserved in S. aureus and whether this might contribute to DAP resistance (Dap^R^). Analysis of LTA production in a methicillin-sensitive S. aureus *mprF* mutant showed that the LTAs migrated faster than those of wild-type strain SA113 (Fig. 6A and Fig. S10A). However, when exposed to daptomycin, the length and production of LTAs increased in both S. aureus Δ*mprF* and wild-type strains (samples treated with 0.25 and 0.5 μg/mL DAP, respectively) (Fig. S10B and C). It is also noteworthy that even when exposed to DAP, the LTAs produced by the wild type remained longer than those of the Δ*mprF* mutant, which perhaps indicates that the LTA length plays a role in DAP sensitivity. This effect was observed at DAP concentrations that did not significantly impact wild-type growth (Fig. S10E) but significantly impaired the growth of the *mprF* mutant (Fig. S10D and E), as previously reported (52, 53). These results are consistent with the idea that the role of MprF in modulating LTA production is conserved in both *Bacillus* and Staphylococcus species, with a change in the nature and/or production of LTAs (rather than an alteration in membrane properties) being associated with DAP resistance.

10.1128/mbio.02667-22.10 FIG S10 Effects of Ca^2+^-daptomycin on growth and LTA production in B. subtilis and S. aureus. (A) Samples of methicillin-sensitive Staphylococcus aureus strains SA113 and SA113 Δ*mprF* (Table 2) were probed for LTA by Western blotting. Three independent sets of cultures grown in LB medium at 37°C were tested simultaneously. (B) S. aureus strains SA113 and SA113 Δ*mprF* were initially grown exponentially for 3 h, after which calcium-daptomycin (DAP) or calcium only (1.25 mM final concentration) was added to the culture for an hour. The collected samples were analyzed by LTA Western blotting as described above for panel A. (C) Analysis of the blot image obtained in panel B processed in Fiji (ImageJ) using the gel analysis function to extract the LTA signal for each sample. (D) The growth of strains SA113 and SA113 Δ*mprF* in the presence of calcium-daptomycin was monitored using a plate reader. Here, cells were diluted at an OD_600_ of 0.05 in LB medium using a preculture of cells in the exponential growth phase. Graphs represent averages of triplicate values. (E) Growth of strains exposed to a range of calcium-daptomycin concentrations similar to those used in panel B when LTA changes were observed. Growth was monitored using a plate reader. Graphs represent the averages of triplicate values. (F) Detection of LTA in daptomycin-resistant strains of B. subtilis. The B. subtilis Dap^R^1 and Dap^R^20 mutants, carrying the *pgsA^A64V^* allele conferring daptomycin resistance (Table 2), and strains 168 1A1 (wild type [WT]), HB15516 (Dap^R^1 *pgsA*^WT^), and HB15507 (Dap^R^20 *pgsA*^WT^) were grown in NB overnight at 30°C and to the late exponential growth phase (37°C, using these cultures grown overnight). For each set of samples, LTA was detected along with PBP2B production by Western blotting. The bottom panel shows the cellular abundances of PBP2B and the LTA synthase detected with anti-LtaS antibody (which cross-reacts with YfnI [Errington, unpublished]). Samples used in the bottom panel were those from the LTA/PBP2B experiment loaded onto a new SDS gel after sample normalization based on the PBP2B signal shown in the top panel. (G) Growth curves for the B. subtilis 168CA, Δ*mprF* (AG1663), Δ*dlt* (DLT71CA), Δ*ltaS* Δ*yfnI* Δ*yqgS* (AG600), Δ*yqgS* Δ*yfnI* (AG595), Δ*ltaS* Δ*yqgS* (AG593), and Δ*ltaS* Δ*yfnI* (AG594) strains in NB at 37°C in the presence of calcium-daptomycin, monitored using a plate reader. Graphs are representative of the results from 1 set out of 3 independent experiments. Download FIG S10, TIF file, 0.7 MB. Copyright © 2023 Guyet et al. 2023 Guyet et al. https://creativecommons.org/licenses/by/4.0/ This content is distributed under the terms of the Creative Commons Attribution 4.0 International license.

As we could not test this idea directly using Dap^R^ MRSA strains due to laboratory regulations, we turned our interest to the strongest known Dap^R^ allele in B. subtilis, *pgsA^A64V^* (54). This mutation of the essential gene *pgsA*, encoding the PGol synthase (Fig. 1B), has been shown to reduce cellular PGol contents and was suggested to modify LTA production (54). Thus, we compared the LTAs of B. subtilis 168 1A1 (WT), isogenic strains carrying *pgsA^A64V^* (strains Dap^R^1 and Dap^R^20), and the *pgsA^A64V^* strain complemented with *pgsA*^WT^ grown overnight in NB. As shown in Fig. 6B and Fig. S10F, the LTA signal below the 30-kDa marker was essentially absent in the Dap^R^ strains, whereas the *pgsA*-complemented strains (Dap^R^ *pgsA*^WT^) had LTA profiles similar to that of the wild-type strain. Thus, PgsA^A64V^ accounts for the severe reduction in LTA production. Finally, we verified that the two major LTA synthases were still produced in the Dap^R^ strains by Western blotting (Fig. 6B and Fig. S10F). A strong LtaS/YfnI signal was detected in the Dap^R^ strains, which proved that one or both LTA synthases were still expressed but upregulated.

The above-described findings in S. aureus and B. subtilis suggested that alterations in LTAs could be associated with DAP sensitivity. As S. aureus *ltaS* is essential, we analyzed the DAP sensitivity of B. subtilis expressing only one LTA synthase. Our assays on NA (Fig. 6C) and NB (Fig. S10G) showed that strains lacking the major LTA synthase LtaS were sensitive to DAP, and this sensitivity was higher than that observed for the Δ*mprF* or Δ*dlt* mutant (Fig. S10G). Thus, LtaS activity is required for DAP tolerance in B. subtilis, more so than MprF.

## DISCUSSION

**FIG 7:**
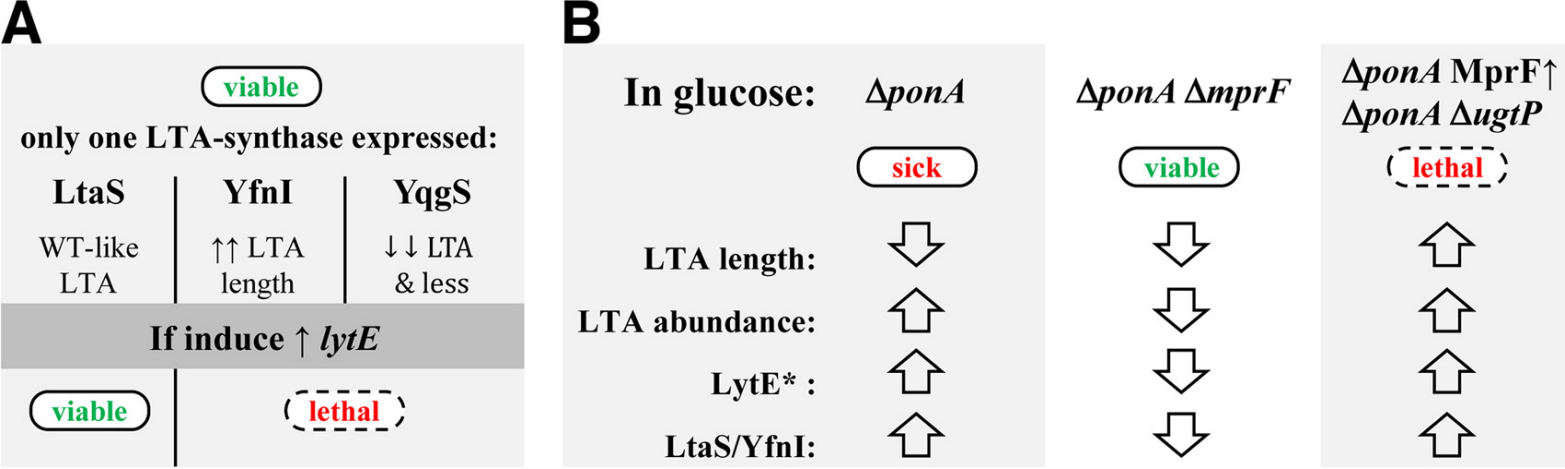
Summary of the alterations in LTA and LytE levels associated with the conditional lethality of the Δ*ltaS* and Δ*ponA* mutants. (A) The overexpression of *lytE* (inducible) in the LTA synthase double mutants is lethal except when the major LTA synthase LtaS is functional. The presence of a distinct LTA length is presented by the mutant strains (Fig. 4A and Fig. 5A). (B) In glucose, the viability and lethality of mutants in the Δ*ponA* background were associated with distinct LTA, LytE, and LtaS/YfnI levels (Fig. 2A and B, Fig. 3A, Fig. 4B and C, and Fig. 5A, C, and D). * indicates that the overexpression of *lytE* (inducible) is lethal to the Δ*ponA* mutant but not the Δ*ponA* Δ*mprF* mutant (Fig. 5A). Strains with the ability to grow without significant delays are referred to as viable (green text in the cell). Lethal refers to the absence of the growth of the mutant on NA or under the specified conditions (dashed cell with red text). In these cases, analysis of the strains was done by shifting cultures to nonpermissive conditions (e.g., low magnesium and the addition of glucose or an inducer). Arrows indicate an increase (↑) or a decrease (↓) in the specified factor. Here, it is assumed that the strength of the LTA signal observed by Western blotting is proportional to the abundance.

**FIG 8:**
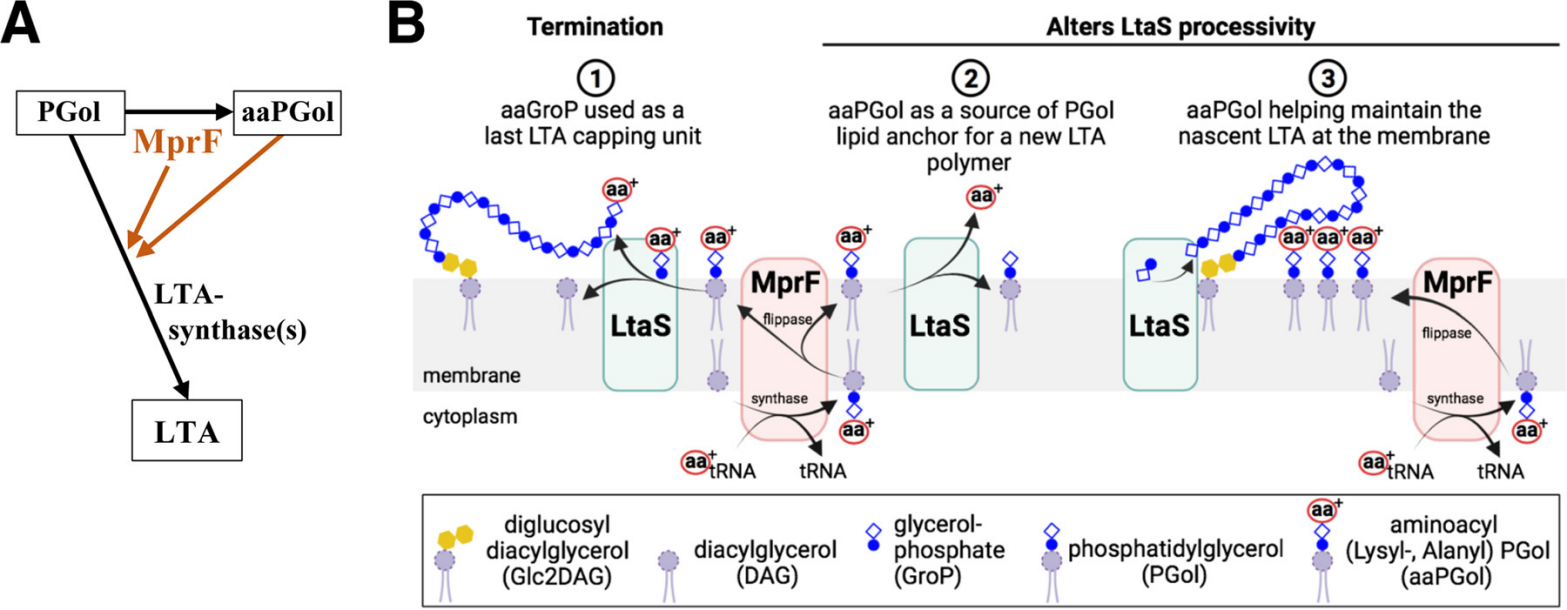
MprF positively regulates the synthesis of LTA in B. subtilis and S. aureus. (A) MprF or its substrates positively regulate LTA production in B. subtilis and S. aureus. (B) Proposed role of MprF in LTA production via its substrate aaPGol (not limiting). (1) LtaS could use the aminoacyl-glycerolphosphate (aaGroP) of aaPGol as the last LTA capping unit. Potentially, the addition of this positively charged capping unit (e.g., lysyl- and alanyl-GroP) could contribute to protection against CAMPs. (2 and 3) MprF indirectly alters LtaS processivity. The release of the aminoacyl could permit LtaS to use the PGol as a lipid anchor (2), and the presence of aaPGol in the vicinity of LtaS could create an electrostatic environment that helps maintain the growing LTA chain in a conformation that favors LtaS processing (3). (Illustration created with BioRender.com.)

In the absence of the class A PBPs (Δ*4*), B. subtilis is viable due to the presence of RodA, the essential PG glycosyltransferase (controlled at least in part by the cell envelope stress regulator σ^M^ [11, 12]), and yet lethal on glucose-rich medium (Fig. 2A; see also Fig. S1B in the supplemental material). By studying either Δ*4* or the Δ*ponA* single mutant, we now understand that the presence of glucose leads to increases in the abundances of the LTA synthases LtaS and YfnI. The increases in these enzymes result in altered LTA production, which in turn causes the accumulation of the major autolysin LytE (Fig. 4 and 5), resulting in an inability to regulate the activity of autolysins (Fig. 7). This lytic phenotype could be suppressed by the absence of MprF (Fig. 2A and Fig. S1B), and we present evidence that MprF is involved in regulating LTA production (Fig. 8). In the Δ*ponA* mutant, the loss of MprF resulted in reduced LTA production and decreased levels of LytE (Fig. 4B, Fig. 5C, and Fig. S5D). This was consistent with our finding that increases in the length and abundance of LTAs (overexpression of *mprF* and Δ*ugtP* mutants) and/or an increase in LytE correlated with glucose-mediated lethality in the Δ*ponA* mutant (Fig. 2B, Fig. 3A, Fig. 4B and C, Fig. 5A, Fig. S5D, and Fig. S9B and C). However, the loss of the major LTA synthase LtaS improved the growth of the Δ*4* mutant only slightly (Fig. 3B and Fig. S1B) but did not significantly improve that of the Δ*ponA* mutant (Fig. S1B). We assume that this was because the loss of LtaS led to the production of very long LTA polymers and a persistently high level of LytE (Fig. 4C and Fig. 5C). In support of this, the analysis of strains defective only in LTA synthesis (last three rows in Fig. 5A) indicated that LtaS is required to regulate *lytE* when artificially overexpressed (Fig. 5A and Fig. 7), presumably because a distinct LTA length range (7 to 17 kDa) and/or an abundance similar that of the wild type (Fig. 4A) is required to counteract excess LytE activity (Fig. 7).

The suggestion that LTAs could inhibit the activities of autolysins was proposed many years ago (1, 5), but the key factors involved have not been identified. Recent work in S. aureus showed that a Δ*ugtP* mutant producing long LTAs is susceptible to PG hydrolases (55), and it has been suggested that the major autolysin LytA is sequestered by LTAs during exponential growth (56). Our results indicate that LytE in B. subtilis exhibits similar characteristics and that the length of LTAs has a role in modulating the abundance of LytE or, presumably, its activity (Fig. 7). We still do not know how this mechanism may work in relation to the location of the LTAs in the wall relative to the active autolysins, particularly LytE, and this remains an important question to be answered in future work.

In B. subtilis 168, the expression of *lytE* is quite complex and coordinated by regulators such as σ^I^ and WalR (46, 57). In contrast, the transcriptional regulation of *mprF* is not well defined, although analyses of another B. subtilis wild-type strain, BSB1, have shown that the expression of *mprF* and *pgsA* is positively correlated with those of *walKR*, the essential two-component response system that controls cell wall metabolism, and, notably, *lytE* (cluster A418 in the *Subti*Wiki expression data browser [58, 59]). However, the expression of *walKR* and *sigI* is not significantly altered in Luria-Bertani medium containing glucose (LB-glucose) (58) or in the absence of all aPBPs (12). Possibly, these regulators play a posttranscriptional role in the Δ*4* mutant where the σ^M^ cell wall stress response is known to be active (12, 57). Some of the cell wall-associated genes mentioned previously (*sigM*, *ponA*, *ltaS*, *yfnI*, *mprF*, and *pgsA*) are differently expressed in cells of B. subtilis BSB1 in LB-glucose medium (58, 59), suggesting that B. subtilis needs to adapt its cell wall metabolism in response to glucose. This might account for the partial redundancy of the functions of the B. subtilis LTA synthases. Evidence also suggests that the presence of magnesium in culture medium leads *Bacillus* cells to adjust their cell wall metabolism. When investigating the effect of magnesium on aPBP mutants in the presence of glucose, we have observed that magnesium decreased the abundance of LytE significantly in both the Δ*ponA* and Δ*ponA* Δ*mprF* mutants (Fig. S9) and less significantly in the Δ*ponA* Δ*ltaS* mutant. In addition, high levels of magnesium have an impact on LTA production (our work in progress) and other processes (60, 61). Thus, the mechanism by which exogenous magnesium improves the growth of some cell wall mutants might require LtaS activity to tune the abundance of LytE in the cell wall (14). Altogether, the results of our study bring a new perspective on the importance of LTAs for cell envelope stability and how extracellular biosynthesis is regulated.

MprF is known to synthesize aminoacyl-phosphatidylglycerol (aaPGol) phospholipids and translocate them across the membrane (Fig. 1). In this work, we show that MprF activity impacts the biosynthesis of LTAs (Fig. 8). The LTA profiles observed in B. subtilis and S. aureus lacking MprF (seen as being shorter and/or having decreased abundances) and the overexpression of B. subtilis MprF (causing smeared and retarded mobility of the LTAs) (Fig. 4B and Fig. 6A) suggest that MprF positively regulates LTA production (Fig. 8A). Other studies have indicated that MprFs may have relaxed substrate specificities across species (22, 62) and even synthesize lysyl-glucosyl-DAG glycolipid in Streptococcus agalactiae (63). Recently, it has been shown that when S. aureus LtaS uses PGol as an alternative anchor for the glycolipid produced by UgtP, the concentrations of these free lipid starter units regulate the length of the LTA polymerized *in vitro* (33). We do not yet know how LTA synthesis is regulated by MprF, but it seems to be LtaS dependent. It is possible that MprF provides the last capping unit (Lys- and Ala-GroP) for LtaS to terminate the LTA being polymerized; this would also contribute to protection against CAMPs (Fig. 8B1). Alternatively, aaPGols might be a source of GroP units (Fig. 8B2) for the formation of LTAs in addition to the known PGol source altering processivity (33). Another possibility would be that aaPGols change the electrostatic environment and that this impacts the activity of the LTA synthase(s) by keeping the LTA polymers adjacent to the membrane (Fig. 8B3).

Gain-of-function mutations in MprF have been identified in methicillin-resistant S. aureus (MRSA) clinical isolates that have acquired resistance to daptomycin (DAP), an antibiotic used as a last resort to treat MRSA infections (26, 53). Over the years, MprF-mediated resistance to DAP (Dap^R^) has been investigated, but a consensus model has yet to link Dap^R^ to a particular domain of MprF and/or to a particular LysPGol content in one or both membrane leaflets (26, 64). We showed that the B. subtilis Dap^R^ *pgsA^A64V^* mutant, known to have a reduced PGol content (54), has a significant decrease in LTAs (Fig. 6B) and that the LTA synthase LtaS is required for the DAP tolerance of B. subtilis (Fig. 6C). Together, the results of this study lead to a new model where modifications of LTA synthesis and/or polymer composition through the modulation of LTA synthesis via MprF combined with changes in the d-alanylation of teichoic acid (65, 66) and phospholipid composition (50) result in a less permeable cell envelope. Thus, DAP resistance would not necessarily be associated with an increase in cell wall thickness (67). Analyses of LTA synthesis and the cell wall composition of MRSA clinical isolates will help verify our MprF-mediated Dap^R^ mechanism and help appreciate the role of MprF in the net cell surface charge and protection against cationic antimicrobial peptides. Our results also give support to the practical application of compounds targeting LTA synthesis (68, 69) and MprF-targeting monoclonal antibodies (70) as approaches to treat clinically relevant bacteria.

## MATERIALS AND METHODS

### Plasmids, bacterial strains, and primers.

The plasmids and strains used in this study are listed in Tables 1 and 2. Strain construction is described below and in Table 2 (last column). The primers used are listed in Table 3.

### Growth conditions and general methods.

B. subtilis strains were grown routinely on nutrient agar (NA; Oxoid) or in nutrient broth (NB; Oxoid). For some assays, strains were grown in liquid Difco antibiotic medium 3 (Penassay broth [PAB]; Oxoid) and on PAB agar (15 g/L agar; Oxoid). B. subtilis transformations were carried out as previously described using SMM defined minimal medium (71, 72). B. subtilis cultures were usually incubated at 30°C (overnight), and the next morning, they were diluted and grown at 37°C (unless otherwise specified). Escherichia coli and Staphylococcus aureus strains were incubated at 37°C on NA plates or in Luria-Bertani (LB) medium. PCR, plasmid manipulations, and E. coli transformation were carried out using standard methods. Strains were selected on NA supplemented with ampicillin (100 μg/mL for E. coli), spectinomycin (60 μg/mL), kanamycin (5 μg/mL), phleomycin (1 μg/mL), erythromycin (0.5 μg/mL), chloramphenicol (5 μg/mL), or daptomycin (see below). Various supplements were also used: IPTG (isopropyl-β-d-thiogalactopyranoside) (0.1 or 1 mM), X-Gal (5-bromo-4-chloro-3-indolyl-β-d-galactopyranoside) (100 μg/mL), MgSO_4_ (between 5 mM and 25 mM as required), and glucose (final concentrations of up to 1%). For NA plates supplemented with 20 mM MgSO_4_, the concentrations of spectinomycin, kanamycin, and erythromycin were doubled to avoid the growth of false-positive strains. Strains with the pMUTin*-his* integration were always grown in the presence of erythromycin.

### Plasmid construction.

To obtain the pDR111*-P_hyspank_-mprF* plasmid, the *mprF* (BSU_08425) coding sequence was amplified using primers oAG271 and oAG272, which changed the start codon from TTG to ATG. The PCR product and the pDR111 plasmid (Table 1) were digested with the SalI and SphI restriction enzymes, ligated, and transformed into E. coli DH5α. Digestion and PCR sequencing using primers oAG300 and oAG301 verified the presence of the insert.

To construct pDR111-*P_hyspank_*-*ltaS*, pDR111-*P_hyspank_*-*yfnI*, and pDR111-*P_hyspank_*-*yqgS*, each LTA synthase gene was amplified by PCR with primer pairs oAG308-oAG309 (2,064 bp), oAG310-oAG311 (2,078 bp), and oAG312-oAG313 (1,990 bp), respectively. PCR products were digested with *Nhe*I and *Sph*I, each ligated to pDR111 digested with the same restriction enzymes. The next steps were carried out as described above for pDR111*-P_hyspank_-mprF*.

For pLOSS-*P_spac_-lytE*, *lytE* was amplified by PCR with primers oAG348 and oAG349 (1,143 bp), and the PCR product was inserted into pLOSS* (Table 1) as *Not*I and *Bam*HI fragments. After transformation, the resulting plasmid was checked by PCRs with primers oAG18 and oAG19 and finally checked by sequencing.

To construct the plasmids pMUTin-′*lytE-his*, pMUTin-′*ltaS-his*, and pMUTin-′*yfnI-his*, the 3′-terminal part of the gene of interest (except for the stop codon) was amplified with primer pairs oAG354-oAG355, oAG366-oAG367, and oAG360-oAG361, respectively, to obtain PCR products ranging from 288 to 309 bp. The PCR products and plasmid pMUTinHis (Table 1) (73) were digested with *Eco*RI and *Xho*I, ligated, and transformed into E. coli DH5α. Each ′*lytE*, ′*ltaS*, and ′*yfnI* insertion was verified by PCR and plasmid sequencing using primers oAG368 and oAG369.

To construct pAM-21, plasmid pET28a(+) (Novagen) and *lytE* (from bp 76, removing the coding sequence for the signal peptide) were amplified by PCRs using primer pairs oAA01-oAA02 and oAA03-oAA04, respectively. The DNA fragments generated were assembled using the NEBuilder HiFi DNA assembly kit (New England BioLabs [NEB]). The resulting plasmid was verified by sequencing using primers oAA05 and oAA06.

### B. subtilis directed mutagenesis.

The steps leading to the construction of new mutants are indicated in the last column of Table 2, with additional details here. New mutants were often obtained by the transformation of competent cells with clean genomic DNA (gDNA) extracted from another existing B. subtilis mutant. The presence of the mutations was verified by PCR using the “check” primers listed in Table 3.

The construction of some of the markerless class A PBP mutants was previously described (12). Additional mutants described in this study were obtained using plasmids pG^+^host9::Δ*ponA*, pG^+^host10::Δ*pbpD*, and pG^+^host10::Δ*pbpF* (Table 1) (12). PCRs with primers oAG124 to oAG129 were consistently performed to check that the markerless deletions were still present (12).

All transformants of the background strains with, and/or leading to, deletions of *ponA*, *mreB*, *mbl*, *gtaB*, *ugtP*, and *ltaS* were selected on NA medium supplemented with MgSO_4_ (20 to 25 mM) with the appropriate antibiotic.

To construct strain AG181 (Δ*mprF*), DNA fragments upstream and downstream of the *mprF* coding sequence were amplified by PCR using primer pairs oAG103-oAG104 (2,390 bp) and oAG105-oAG106 (2,414 bp), respectively, and digested with *Eco*RI and *Xba*I, respectively. The phleomycin resistance antibiotic cassette was amplified by PCR with primers PhleoXbaI-fw and PhleoEcoRI-rev from plasmid pIC22 and digested with both *Eco*RI and *Xba*I. The 3 digested PCR fragments were ligated to generate a linear DNA and then transformed into 168CA with selection for phleomycin resistance. The deletion of *mprF* was confirmed by PCR using primers oAG107 and oAG108.

Strain AG304 (*amyE*::*P_hyspank_-mprF spc*) was obtained by the transformation of 168CA with the pDR111-*P_hyspank_*-*mprF* plasmid (Table 1). The correct recombination (double crossover) at *amyE* was confirmed by the absence of amylase activity when grown on NA with 0.2% starch and by PCR (primers oAG300 and oAG301). The same methods were used to confirm the mutation obtained after the transformation of other pDR111 derivatives.

We constructed our own *lytE* deletion mutant (Δ*lytE*::*cat*). For this, regions of genomic DNA upstream and downstream of *lytE* were amplified by PCRs with primer pairs oAG350-oAG351 (2,048 bp) and oAG352-oAG353 (2,500 bp) and then digested with *Xba*I and *Bam*HI (Table 3), respectively. The chloramphenicol resistance cassette from the pCotC-GFP plasmid (Table 1) was amplified with primers oAG197 and oAG198. The resulting PCR product (850 bp) was then digested with *Xba*I and *Bam*HI, and the three DNA fragments were ligated and transformed into B. subtilis 168CA. Strain AG547 was obtained from this transformation, and the deletion of *lytE* was confirmed by PCR using primers oAG340 and oAG341 (Table 3).

Strains AG565 and AG586 were obtained by the integration of pMUTin-′*lytE-his* (erythromycin selection) into the chromosomes of 168CA and the Δ*cwlO* mutant (strain AG474), respectively. The plasmid integration (single crossover) in B. subtilis resulted in the expression of the recombinant protein with a C-terminal LEMRGSH_12_ tag under the control of its native promoter. Since the integration of this plasmid into the Δ*cwlO* background did not result in any phenotypic change, this indicated that LytE-His_12_ was functional, as a *lytE cwlO* double mutant is lethal (44). Thereafter, the genomic DNA of strain AG565 was used to transform all of the strains needed for Western blot analysis (Table 2). Of note, strain AG1541 (Δ*cwlO*) obtained by genomic DNA recombination has the same genotype as that of the AG586 strain obtained by plasmid integration.

### Random transposition mutagenesis.

Random transposition mutagenesis was performed as previously described (39, 74) using the pMarB plasmid that carries the transposon Tn*YLB-1* encoding kanamycin resistance. pMarB was introduced into B. subtilis strains carrying pLOSS-*P_spac_*-*ponA* (12) (Table 1), a plasmid with an unstable origin of replication, and *lacZ*, which helps to monitor plasmid stability by observing the formation of blue or white colonies. After screening the random library of mutants, genomic DNAs extracted from positive clones were backcrossed into the parental strain used for the transposon screen. Transposon insertion sites were identified by inverse PCR using gDNA extracted from the backcrossed positive clones (74) and sequencing the resulting PCR products. Further verification of the transposon’s site of insertion was obtained using specific primers.

For the suppressor screen in the *ponA pbpD pbpF* mutant background, we constructed strain AG141 (Table 2), which carries deletions of the three vegetative class A PBPs, the *lacA* gene (74), and pLOSS-*P_spac_*-*ponA* (12). The transposon mutant library was screened on PAB agar supplemented with MgSO_4_ (10 mM), X-Gal, and IPTG (1 mM), and white colonies were selected. Strain SWA11a (Table 2) had a transposon in the *mprF* coding sequence, which was verified using specific primers oAG81 and oAG82 (Table 3).

A conditional lethal screen was also carried out in a *ponA pbpD pbpF mprF lacA* pLOSS-*P_spac_*-*ponA* strain (AG200) (Table 2). Here, the transposon mutant library was screened on NA and PAB supplemented with X-Gal and IPTG (1 mM). Blue colonies, indicating plasmid stability, were selected. One mutant was isolated, and its genomic DNA was backcrossed into the AG200 background. The resulting blue-forming strain, AG200BK#42 (Table 2), was found to carry a transposon in the *gtaB* coding sequence using primers oAG165 and oAG166 (Table 3).

### Spot growth assays.

Strains were grown in NB with 20 mM MgSO_4_ at 30°C overnight. The cultures were then diluted 1:100 into fresh NB with 10 mM MgSO_4_ (or, when applicable, 20 mM MgSO_4_) and grown to mid-exponential phase at 37°C. Cells were harvested, washed in NB, and diluted to an optical density at 600 nm (OD_600_) of ~0.3 in NB. Each culture was then serially diluted 1:10 using a 96-multiwell plate and NB. Using a multichannel pipette, 5 μL of each dilution was transferred to various agar plates. As a control, all of the serial dilutions presented in the figures were also spotted onto media supplemented with 10 mM or 20 mM MgSO_4_ to ensure that growth occurred for all spots (data not shown). Plates were incubated at 37°C overnight and scanned at different time points. Images were edited using Photoshop CS software.

### Microscopy imaging.

Cells were grown to the exponential growth phase at 37°C in NB medium. Before the cells were mounted onto microscope slides, the slides were covered with a thin layer of NA medium supplemented with the FM5-95 membrane dye at 140 μg/mL (Invitrogen). Fluorescence microscopy was carried out with a Zeiss Axiovert 200M microscope attached to a Sony Cool-Snap HQ cooled charge-coupled-device (CCD) camera with a Nikon APO 100×/1.40 oil Ph3 lens objective. Images were acquired using Metamorph 6 imaging software (Molecular Devices, Inc.). Images were analyzed with ImageJ (http://rsb.info.nih.gov/ij/) and assembled with Photoshop CS software.

Images stained with the FM5-95 membrane dye were processed using the ObjectJ plug-in in ImageJ to measure the diameter and length of about 400 cells per strain. Measurements were performed only once as the cell diameter change was obvious under a microscope and in the images in Fig. 3D (Table 4; for details, see Fig. S3A and B in the supplemental material).

### Transmission electron microscopy.

Strains were grown in NB medium (with IPTG when applicable) until they reached an OD_600_ of about 0.4. The cell pellets were fixed in 2% glutaraldehyde in sodium cacodylate buffer (0.1 M) at 4°C for at least 24 h. Samples were spun, molten 4% agarose was added to the samples, and the samples were respun. Samples were allowed to cool in a fridge for 30 min. The agarose block was removed from each tube and cut into 1-mm^3^ pieces. The samples were then dehydrated by microwave processing using the Pelco Biowave Pro+ system incorporating Pelco Coldspot Pro. In a vacuum chamber, samples were rinsed 3 times in 0.1 M sodium cacodylate buffer (150 W for 40 s), treated with 1% osmium, pulsed (100 W for 8 min), and rinsed 3 times in distilled water (150 W for 40 s). They were then taken out of the vacuum chamber, and a graded series of dried acetone was applied to the samples (25%, 50%, 75%, and 3 times with 100% at 150 W for 40 s for each step). This was followed by impregnation with a series of increasing concentrations of epoxy resin (Taab medium resin) in acetone (25%, 50%, 75%, and 3 times with 100% at 300 W for 3 min for each step). Samples were embedded in fresh 100% resin and polymerized at 60°C for 24 h in a conventional oven. Once polymerized, the resin block was cut into semithin survey sections (0.5 μm) and stained with 1% toluidine blue in 1% sodium tetraborate (Borax). Ultrathin sections (approximately 70 nm) were then cut using a diamond knife on a Leica EM UC7 ultramicrotome. The sections were stretched with chloroform to eliminate compression and mounted onto Pioloform-filmed copper grids. The grids were stained with uranyl acetate (2%) and lead citrate and viewed on a Hitachi HT7800 transmission electron microscope using an Emsis Xarosa camera.

### Sample preparation for LTA polymer detection.

B. subtilis strains were grown overnight at 30°C with shaking in NB supplemented with 5 mM MgSO_4_ (or 10 mM MgSO_4_ for *mbl* mutants). The cells were then diluted (1:60 to 1:80 depending on the strain) to an OD_600_ of ~0.02 in prewarmed NB and NB supplemented with 0.2% glucose. The strains were then incubated at 37°C with shaking and grown for about 4.5 h (late exponential phase). At the sampling time, the OD_600_ of the cultures was measured before harvesting 15 to 25 mL of each culture by spinning at 3,273 × *g* for 10 min using a swingout centrifuge at room temperature (RT). Pellets were washed with 200 μL of solution A (with 10 mL of Tris-HCl at 100 mM [pH 7.4] and one cOmplete Mini EDTA-free protease inhibitor cocktail tablet from Roche) and pelleted with a benchtop centrifuge. Cell pellets were then frozen in liquid nitrogen and stored at −20°C until processing. Samples were resuspended in a volume of solution B (an equal volume of solution A and lithium dodecyl sulfate [LDS] sample buffer [Life Technologies]) ranging from 70 to 200 μL in proportion to their OD_600_ values. Samples were boiled for 30 min at 100°C, cooled on ice for 3 min, and incubated with 100 U of Benzonase (catalogue number E1014; Sigma) for 30 min at 37°C. Samples were pelleted at 4°C, and the supernatants containing the extracted LTAs were collected. The supernatants were frozen in liquid nitrogen and kept at −25°C. For LTA Western blotting, the quantity of the sample loaded was normalized using Bio-Rad protein assays such that the final OD_595_ was approximately 0.35. Sample sets to be compared were grown, extracted, measured, and normalized together for each experiment.

S. aureus strains were grown overnight at 37°C with shaking in LB medium. The cells were then diluted (about 300-fold) to an OD_600_ of about 0.02 and grown in LB medium at 37°C until reaching the late exponential growth phase at an OD_600_ of about 1. Ten milliliters of the cultures was harvested, and samples were prepared as described above for B. subtilis, with the following changes: 60 μL of solution B was used, and ~18- to 20-μL samples were loaded onto an SDS-PAGE gel, with samples normalized to their OD_600_ values.

### Total protein sample preparation.

For LytE or LtaS Western blotting, cells were grown, harvested, and processed in the same way as described above for the LTA samples, with the exception that to detect the His-tagged proteins (LytE, LtaS, or YfnI), the strains were grown in the presence of erythromycin. An 8-mL culture sample was resuspended in 180 to 200 μL of solution A and sonicated until a clear lysate was obtained. Samples were centrifuged at 16,000 × *g* for 10 min at 4°C, and a volume of the sample supernatant was loaded onto an SDS gel and normalized based on the OD_600_ of the culture collected.

### SDS-PAGE, LTA and protein Western blotting, and detection blots.

For SDS-PAGE, samples were diluted in solution B with the addition of a reducing agent (1.5 μL per sample) to a final volume of 20 μL, heated at 70°C for 10 min, loaded onto a gel (NuPAGE 4 to 12% Bis-Tris gradient midi gel; Life Technologies), and separated by electrophoresis. For the LTA-PBP2B experiment, morpholineethanesulfonic acid (MES) buffer, a Novex Sharp prestained protein ladder (Life Technologies), and a 0.2-μm Amersham Hybond sequencing polyvinylidene difluoride (PVDF) membrane (GE Healthcare) were used, whereas for LytE (37 kDa) or LtaS (74 kDa) Western blotting, morpholinepropanesulfonic acid (MOPS) buffer, an Abcam prestained midrange protein ladder (catalogue number Ab116027), and a 0.45-μm Amersham Hybond PVDF membrane (GE Healthcare) were used. The SDS-PAGE gels were transferred to membranes using the Trans-Blot Turbo transfer system (Bio-Rad), using an in-house transfer buffer (600 mM Tris, 600 mM glycine, 280 mM Tricine, 2.5 mM EDTA, and 0.05% SDS). After transfer, the membranes were washed three times for 5 min each in phosphate-buffered saline (PBS) using a rocking shaker; afterward, the membranes were cut where required to allow different detection methods.

For LTA Western blotting, the membrane was cut at the 60-kDa ladder position. The bottom part of the membrane was used for LTA blotting, whereas the top part was used to detect the membrane protein PBP2B (79 kDa) as a loading/extraction control. All LTA Western blot steps were performed at room temperature in a rolling shaker using 50-mL Falcon tubes. The LTA membranes were incubated for 1 h 15 min in PBS buffer with 3% bovine serum albumin (BSA) (catalogue number A7030; Sigma) and then for 1 h 30 min in fresh PBS–3% BSA with Gram-positive LTA monoclonal antibody (catalogue number MA1-7402; Thermo Fisher) at a dilution of 1:1,000. The membrane was then washed briefly twice, followed by four 8-min washes in PBS, and then incubated in PBS–5% dried semiskimmed milk with a 1:10,000 dilution of anti-mouse horseradish peroxidase (HRP)-linked secondary antibody (catalogue number A9044; Sigma) for 1 h. This was then followed by two brief washes and six 8-min washes prior to detection.

For Western blot detection of LytE-His_12_ or LtaS and PBP2B, all of the following steps were performed at room temperature using a rocking shaker. The membranes were washed after transfer in PBS–0.1% Tween 20 (PBS-T) and blocked in PBS-T with 5% milk (PBS-T-milk) for 1 h 15 min at room temperature. Thereafter, the LytE-His_12_/PBP2B membrane blot was cut at the ~53-kDa ladder position. Membranes were placed into fresh buffer containing PBS-T-milk for 1 h (or 1 h 30 min when performing the LTA Western blotting in parallel) with polyclonal rabbit anti-PBP2B antibody (at 1:5,000) (laboratory stock or available at Merck [catalogue number ABS2199]), mouse monoclonal penta-His antibody (catalogue number 34660; Qiagen) (stock at 200 μg/mL) (used at a dilution of 1:2,000), or polyclonal rabbit anti-LtaS antibody (at 1:1,000) (laboratory stock). Two brief washing steps were performed in PBS-T, followed by 3 washes for 10 min. Membranes were incubated in PBS-T-milk for 1 h using 1:10,000 dilutions of anti-rabbit or anti-mouse HRP-linked secondary antibody (catalogue number A0545 or A9044, respectively; Sigma) as required. Membranes were then washed as described above for the previous step.

The Pierce ECL Plus Western blotting substrate (catalogue number 32132; Thermo Scientific) was used for detection as recommended by the manufacturer. Chemiluminescence was detected using an ImageQuant LAS 4000 mini digital imaging system (GE Healthcare), and membranes were exposed in 1-min increments for 20 min. Membranes were also imaged by epi-illumination to detect the prestained protein ladder on the membrane. Under our conditions, the quantities of LTA samples loaded were scaled down to avoid burnouts of the signals observed for some of the tested strains. Occasionally, for the LTA blots, strains (in NB only) displayed a very weak signal above the 30-kDa ladder position. This phenomenon was observed by others performing *Bacillus* LTA Western blotting and was assumed to be related not to LTAs but possibly to the cross-reactivity of the antibody to WTAs that also contain glycerolphosphate (18).

Images were edited in ImageJ (Fiji), and the same contrast enhancement was applied to the membranes in the same set of experiments. Three independent sets of experimental samples were generated by Western blotting. Contrast was sometimes added to the images, particularly to observe LytE signals in low-expressing strains.

### LytE purification and raising antiserum.

Plasmid pAM-21 was transferred to the E. coli BL21(DE3) strain, and the culture was grown at 37°C in LB-kanamycin medium. When the culture reached an OD_600_ of ~0.5, *lytE* expression was induced with 1 mM IPTG for 3 h. Fifty milliliters of the culture was collected by centrifugation at 9,000 × *g* for 5 min and resuspended in 15 mL of a solution of PBS with 4 μg/mL of lysozyme. After incubation for 20 min at RT, cells were sonicated, and once a clear lysate was obtained, the cells were harvested at 3,000 × *g* for 4 min. The cell pellet was resuspended in PBS buffer with 8 M urea and incubated for 30 min at RT on a rolling shaker. The cells were then centrifuged at 9,000 × *g* for 10 min, and the supernatant was collected, mixed gently with 200 μL of high-performance Ni-Sepharose (GE Healthcare), and incubated for 1 h under the same conditions as the ones described above. Next, the sample was washed 3 to 4 times in buffer A (50 mM Tris [pH 8] and 300 mM NaCl), and the last wash was done in buffer B (50 mM Tris, 300 mM NaCl, and 5 mM imidazole). The protein was eluted five times in buffer C (PBS with 8 M urea and 100 mM imidazole), and the eluents were analyzed by SDS-PAGE. Ice-cold acetone was added to the desired eluted sample at a 4:1 volume ratio, and the sample was incubated overnight at −20°C. The sample was centrifuged at 4°C for 10 min at 15,000 × *g*, and the pellet was left to dry at RT. The pellet was suspended in 200 μL of PBS, and a 100-μg aliquot was sent for raising antiserum (Eurogentec). The LytE antiserum was used for Western blotting at a 1:5,000 dilution with the anti-rabbit secondary antibody (1:10,000).

### Growth assays in the presence of daptomycin.

For the S. aureus samples for LTA Western blotting, cultures were grown as described above, but after growth in LB medium for 3 h, CaCl_2_ (1.25 mM final concentration) was added to the cultures, which were then divided and grown for an extra hour with or without daptomycin (DAP) (stock at 1 mg/mL) (catalogue number ab141204; Abcam).

For the NA-CaCl_2_-DAP assays, strains were grown in NB for about 3 h at 37°C (exponential phase) and then normalized to an OD_600_ of 0.5. Cells (300 μL) were spread onto 25-mL NA plates supplemented with CaCl_2_ (1.25 mM final concentration). A 0.016- to 256-mg/L daptomycin antimicrobial susceptibility testing strip (Liofilchem) was applied to the surface of the plate. Plates were incubated at 37°C overnight and scanned after 24 h. Images were edited in Fiji (ImageJ). The manufacturer’s guidance was used for the interpretation of growth inhibition.

To monitor strain growth in a plate reader, we used precultures that had been grown in NB (or in LB medium for S. aureus) at 37°C for about 3 h. For each assay, a daptomycin stock at 17.4 mg/mL was used to prepare a fresh DAP stock at 0.1 mg/mL, and this was used to make dilutions of the antibiotic as required in 96-well plates in 100 μL of medium supplemented with CaCl_2_ (1.25 mM final concentration). Plates were then loaded with 100 μL of cells (diluted in medium-CaCl_2_ and normalized to the desired OD_600_). Plates were shaken at 37°C in a Tecan Sunrise plate reader, and the OD_600_ was measured every 6 min. Conditions were tested in duplicates or triplicates whenever possible. Data were collected using Magellan software (version 7.2). Microsoft Excel was used for analysis and the generation of graphs.
