# Supplementary figures and images for: Insights into the Roles of Lipoteichoic Acids and MprF in Bacillus subtilis
Source: mBio. 2023 Feb 6;14(1):e02667-22. doi: 10.1128/mbio.02667-22 (PMC9973362; doi:10.1128/mbio.02667-22)

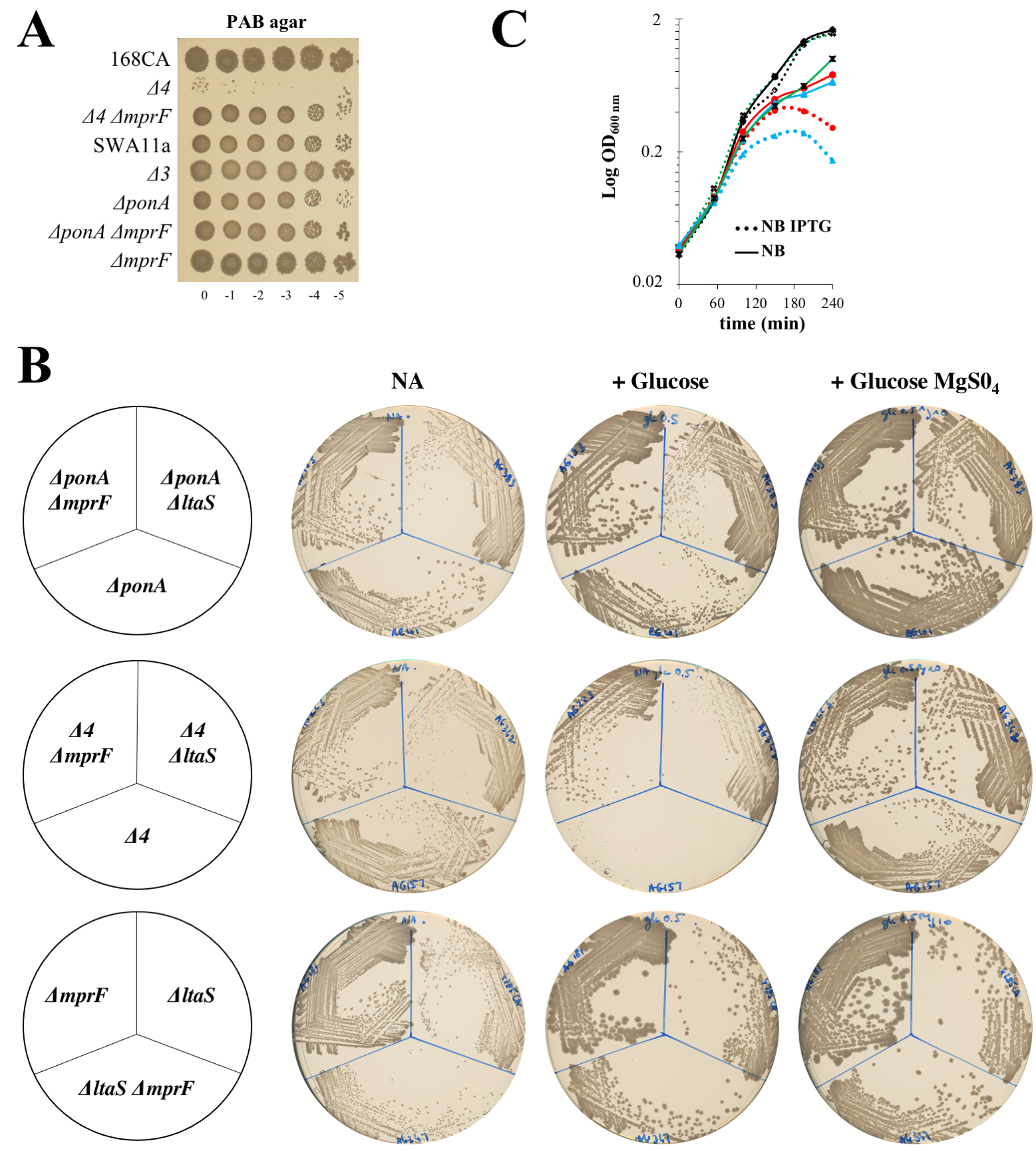

Supplement: FIG S1 [file mbio.02667-22-s0001.tif]

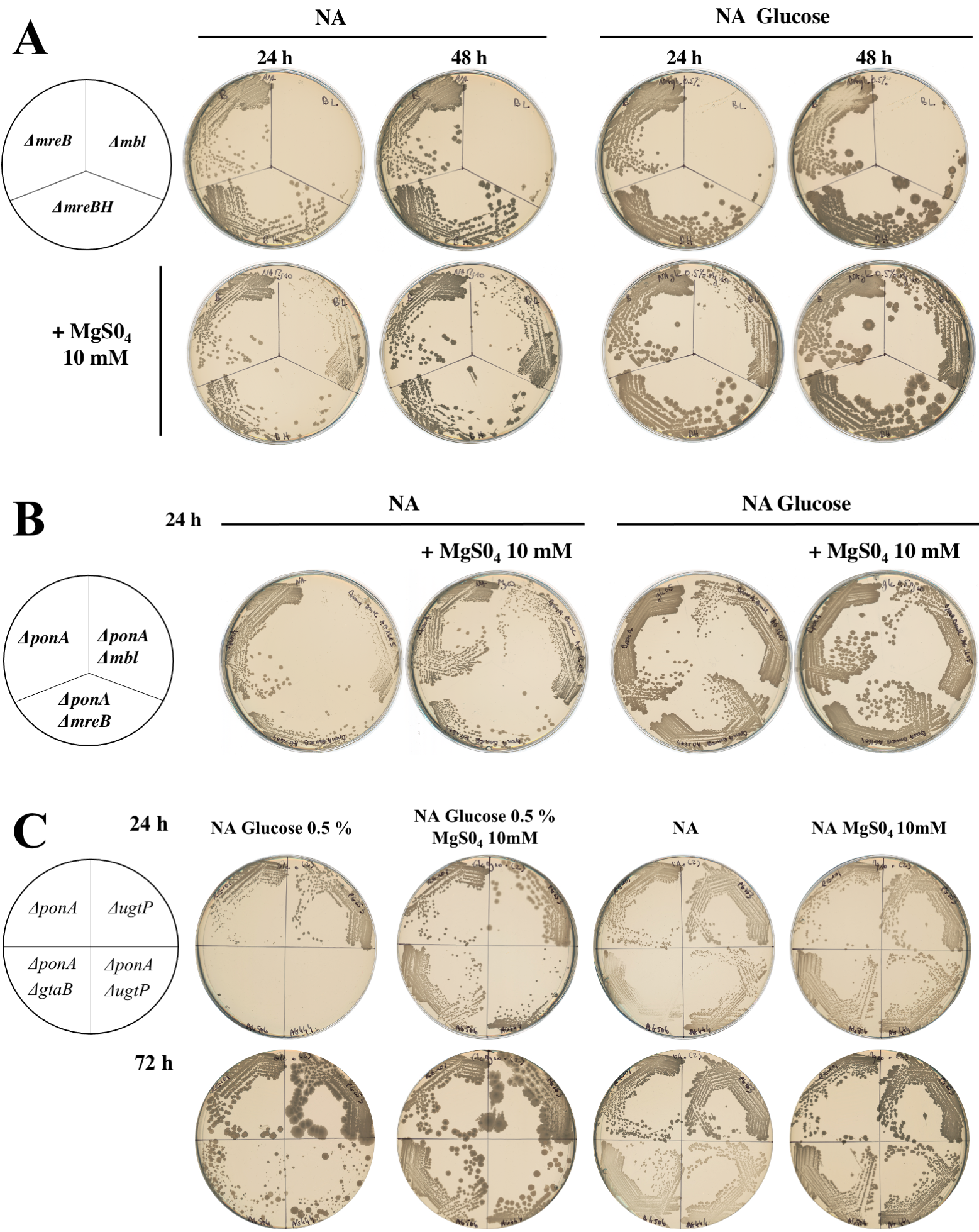

Supplement: FIG S2 [file mbio.02667-22-s0002.tif]

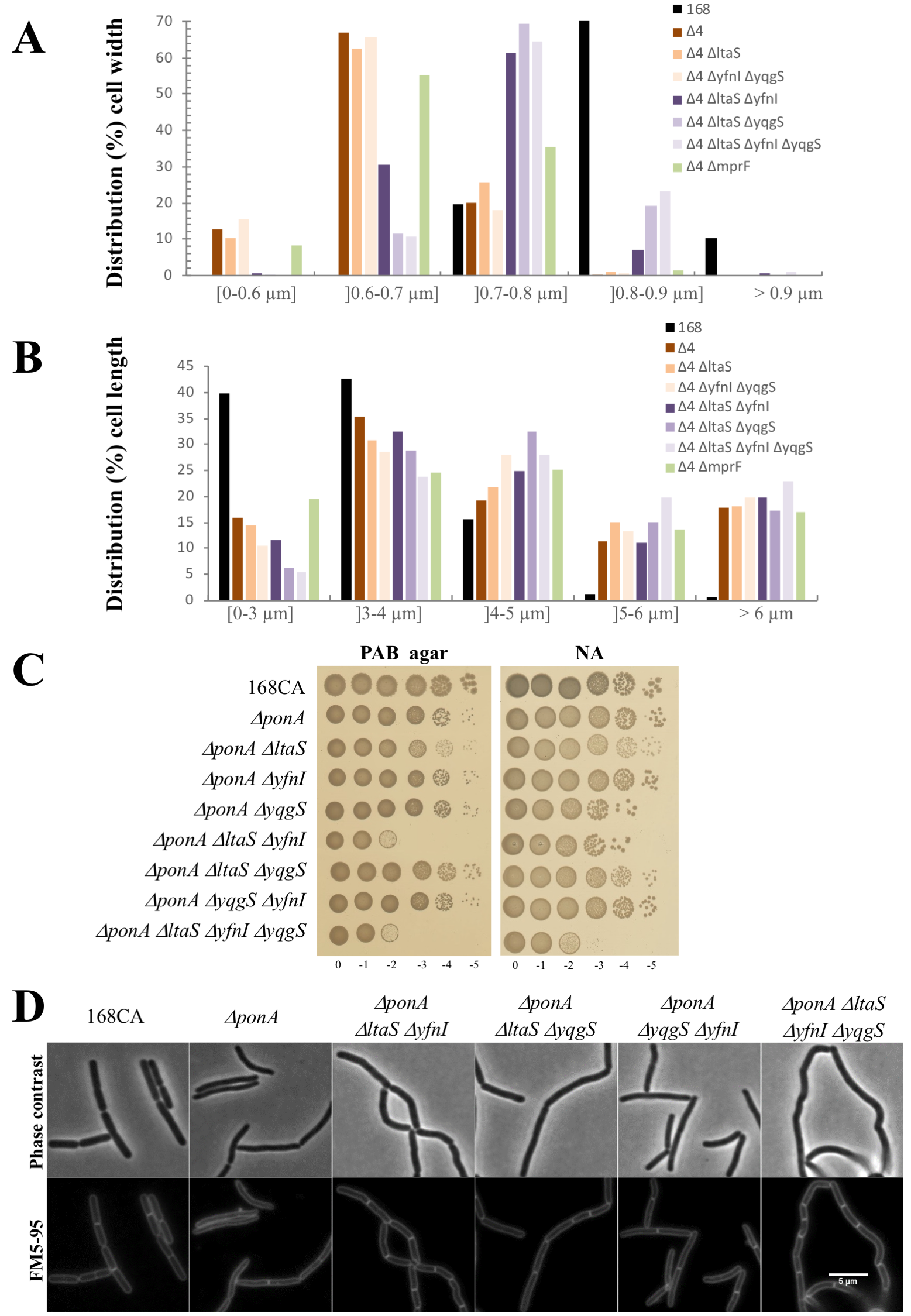

Supplement: FIG S3 [file mbio.02667-22-s0003.tif]

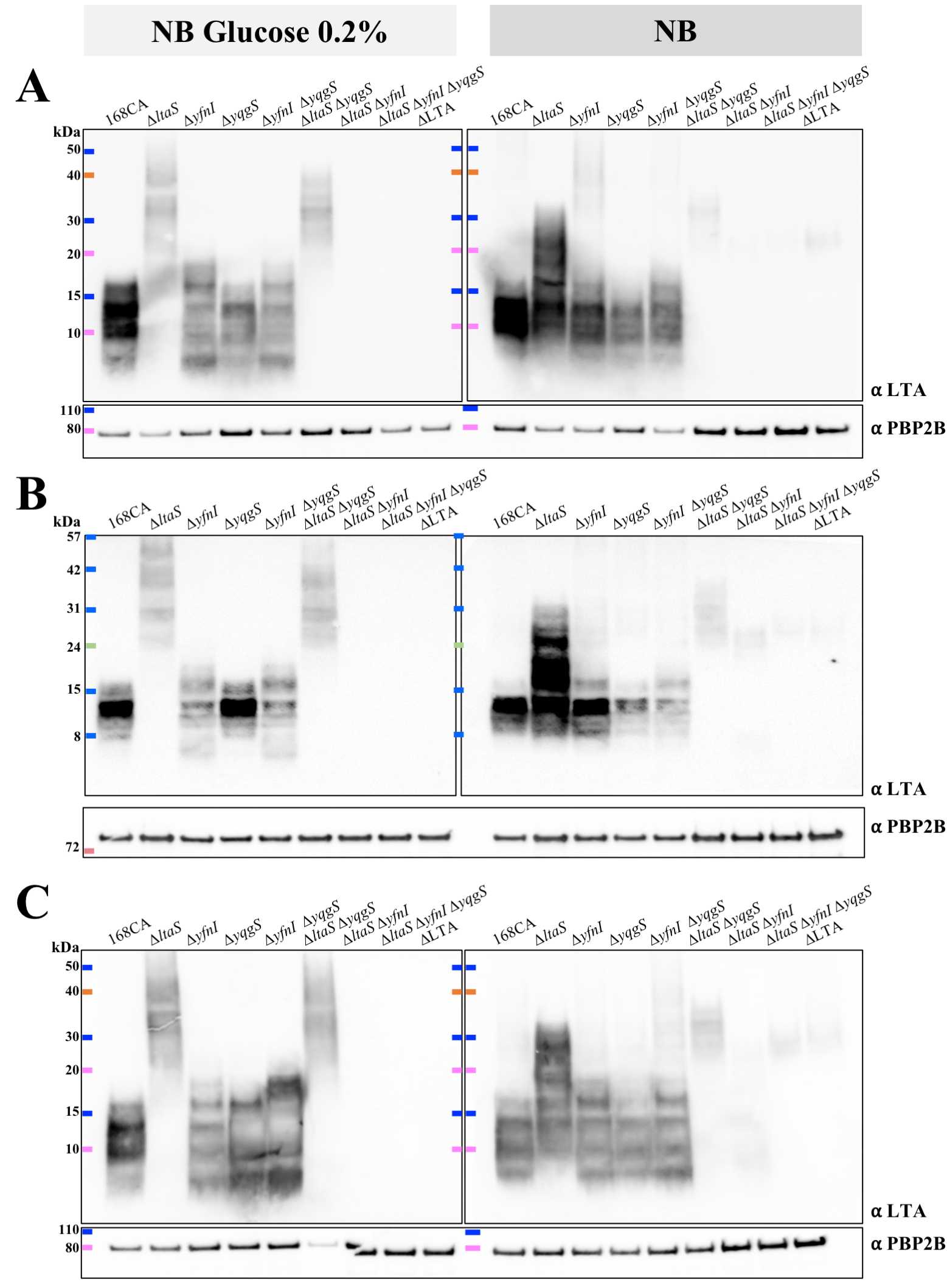

Supplement: FIG S4 [file mbio.02667-22-s0004.tif]

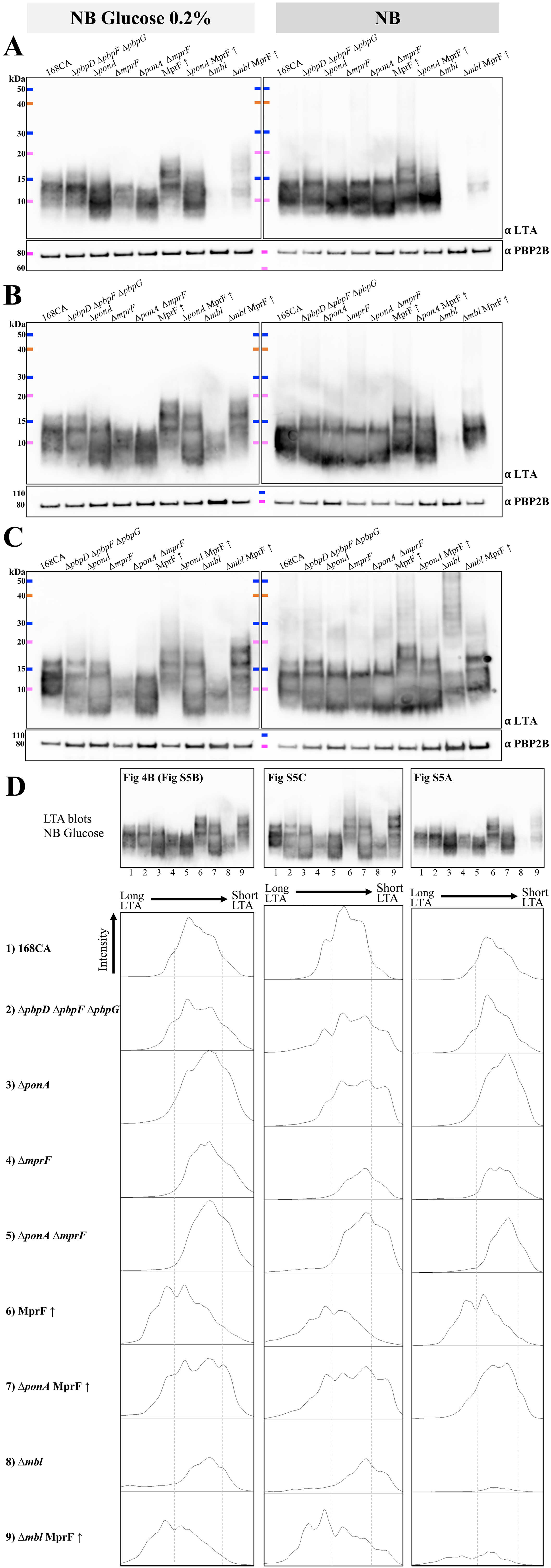

Supplement: FIG S5 [file mbio.02667-22-s0005.tif]

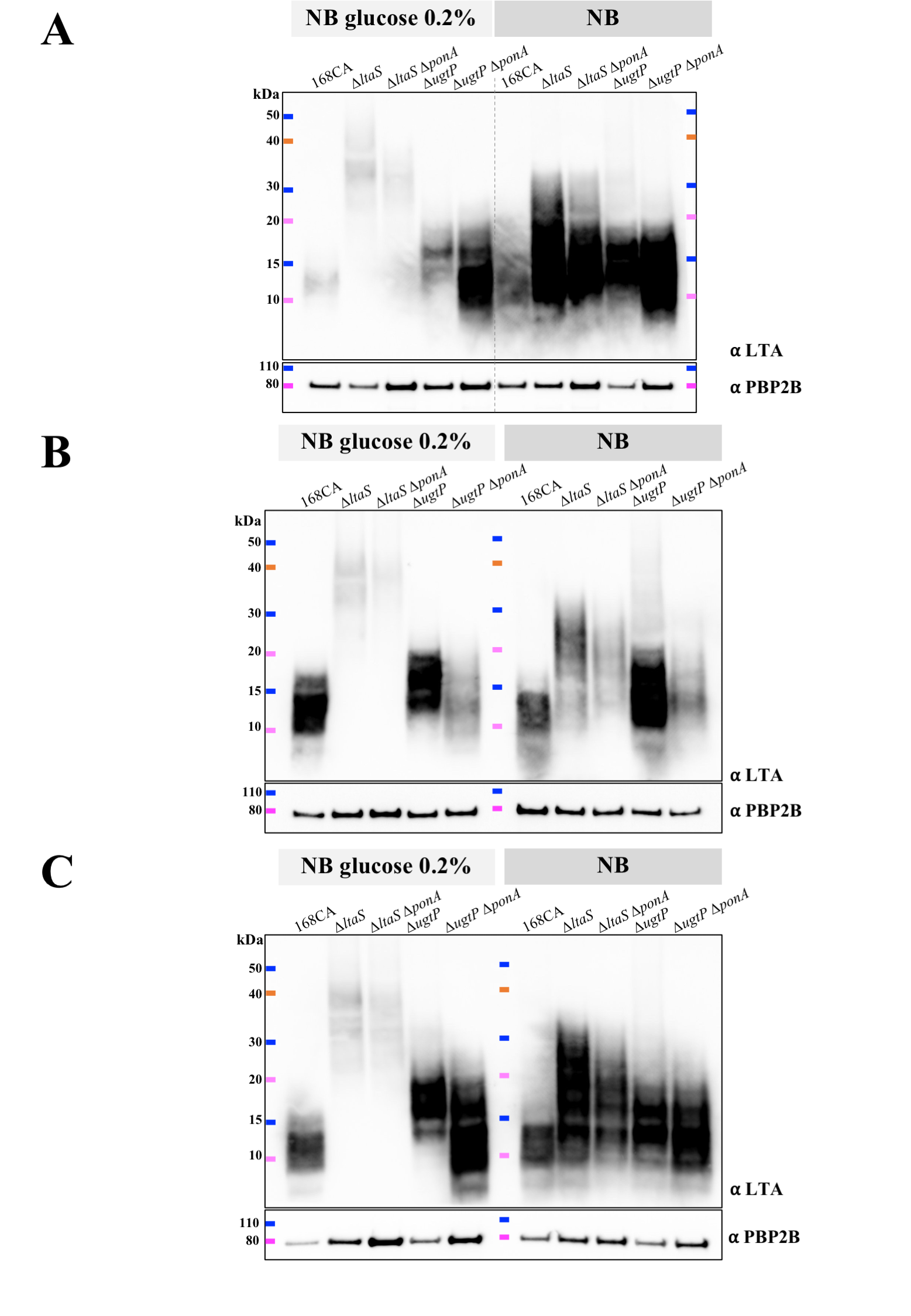

Supplement: FIG S6 [file mbio.02667-22-s0006.tif]

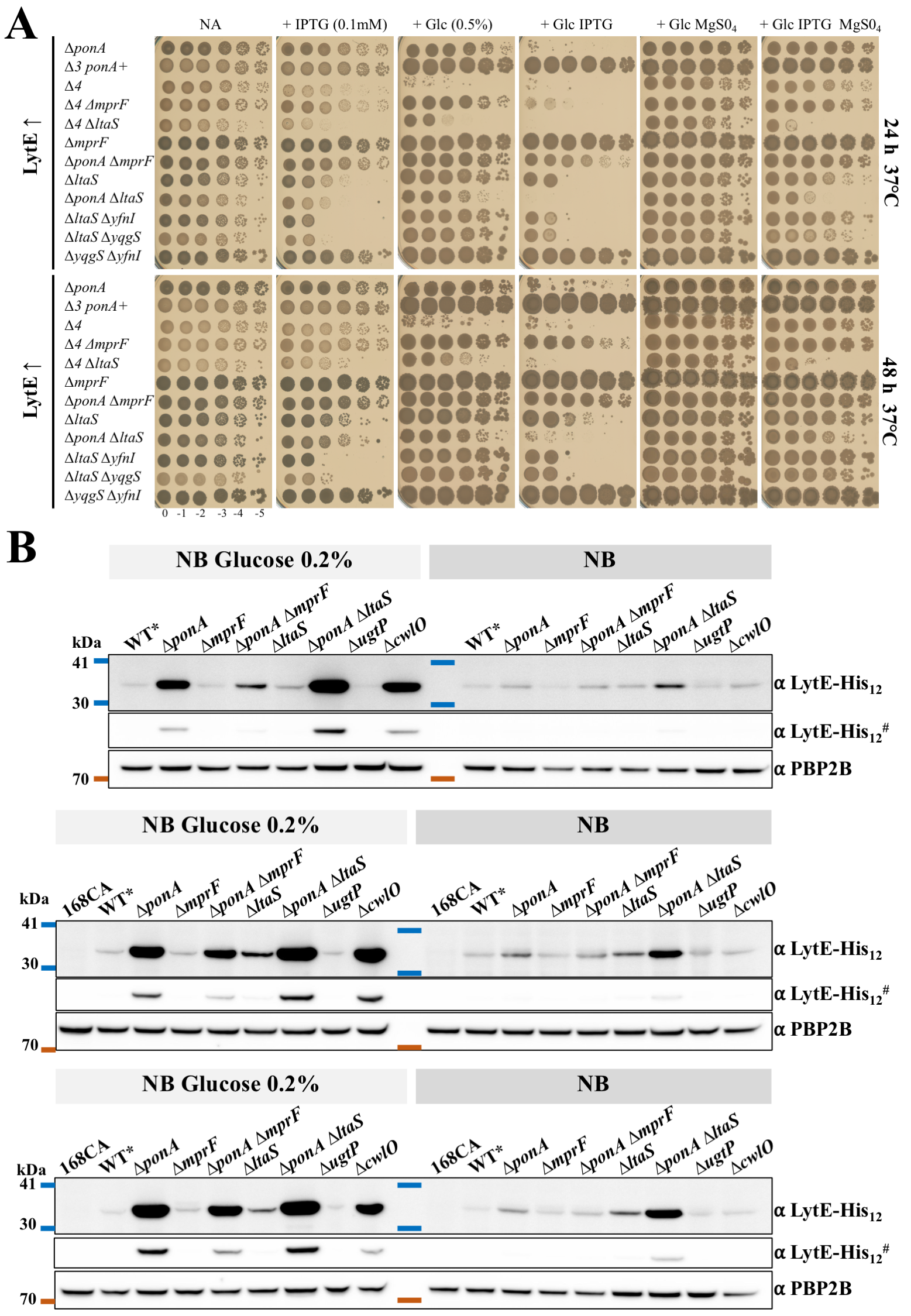

Supplement: FIG S7 [file mbio.02667-22-s0007.tif]

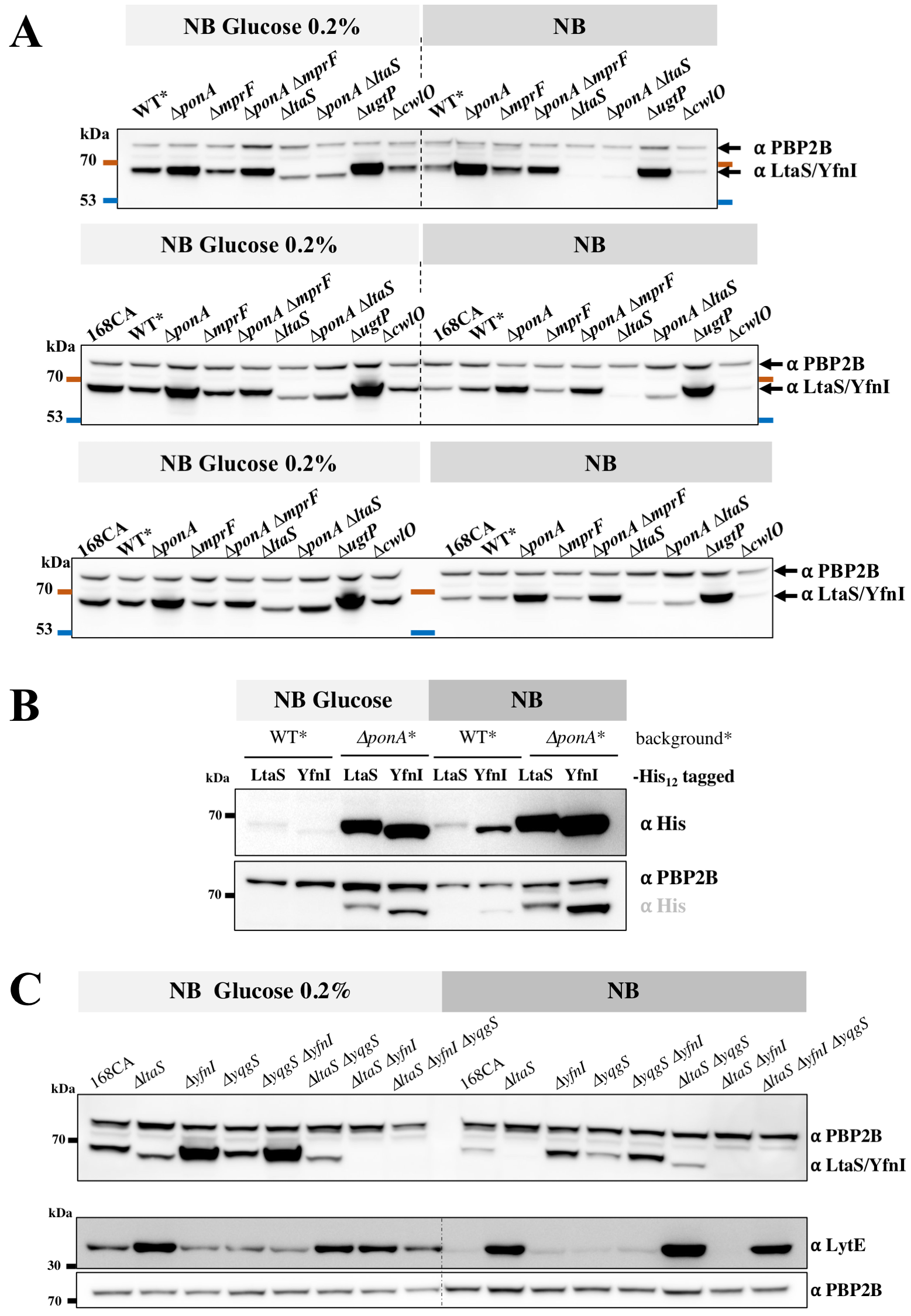

Supplement: FIG S8 [file mbio.02667-22-s0008.tif]

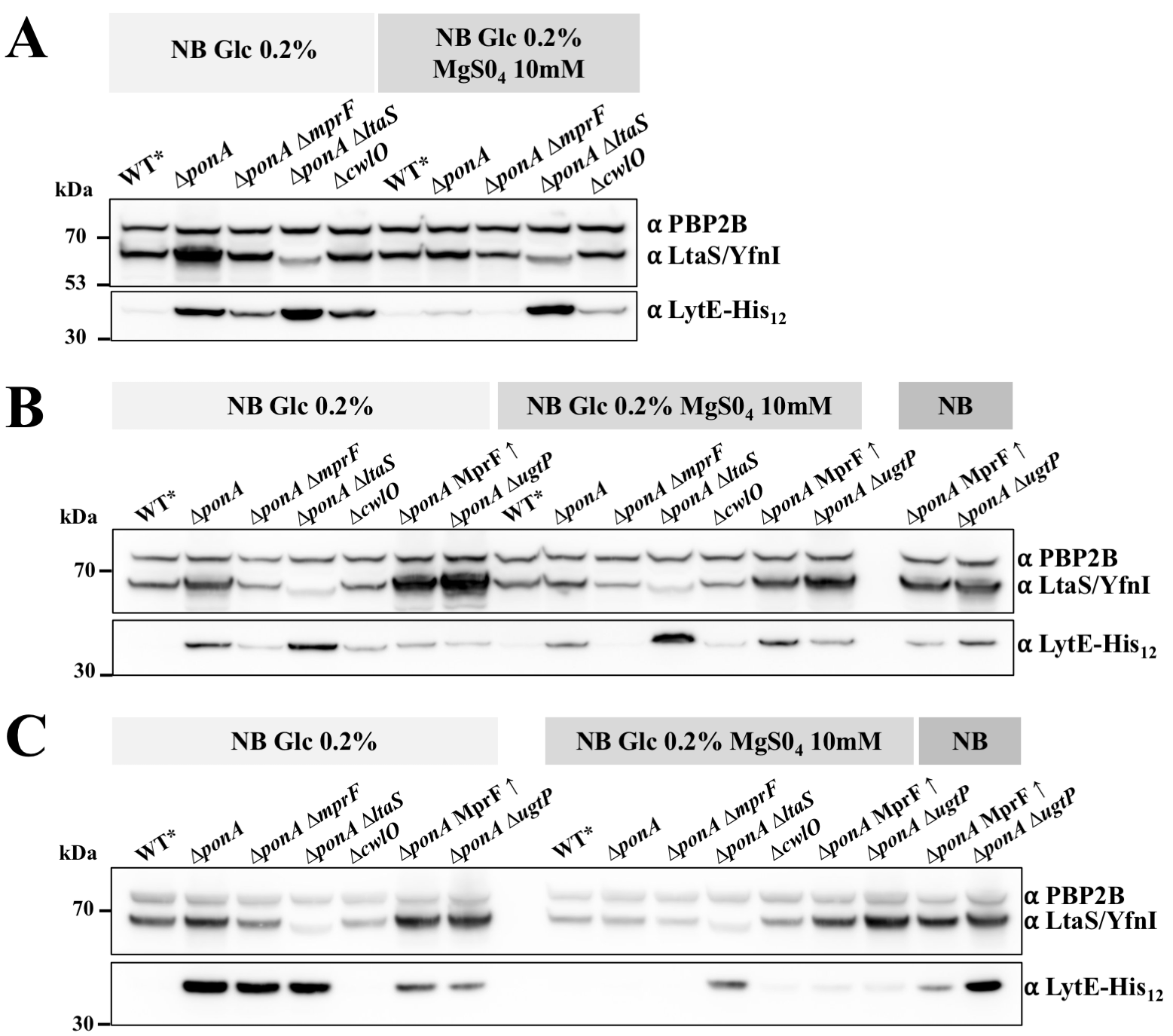

Supplement: FIG S9 [file mbio.02667-22-s0009.tif]

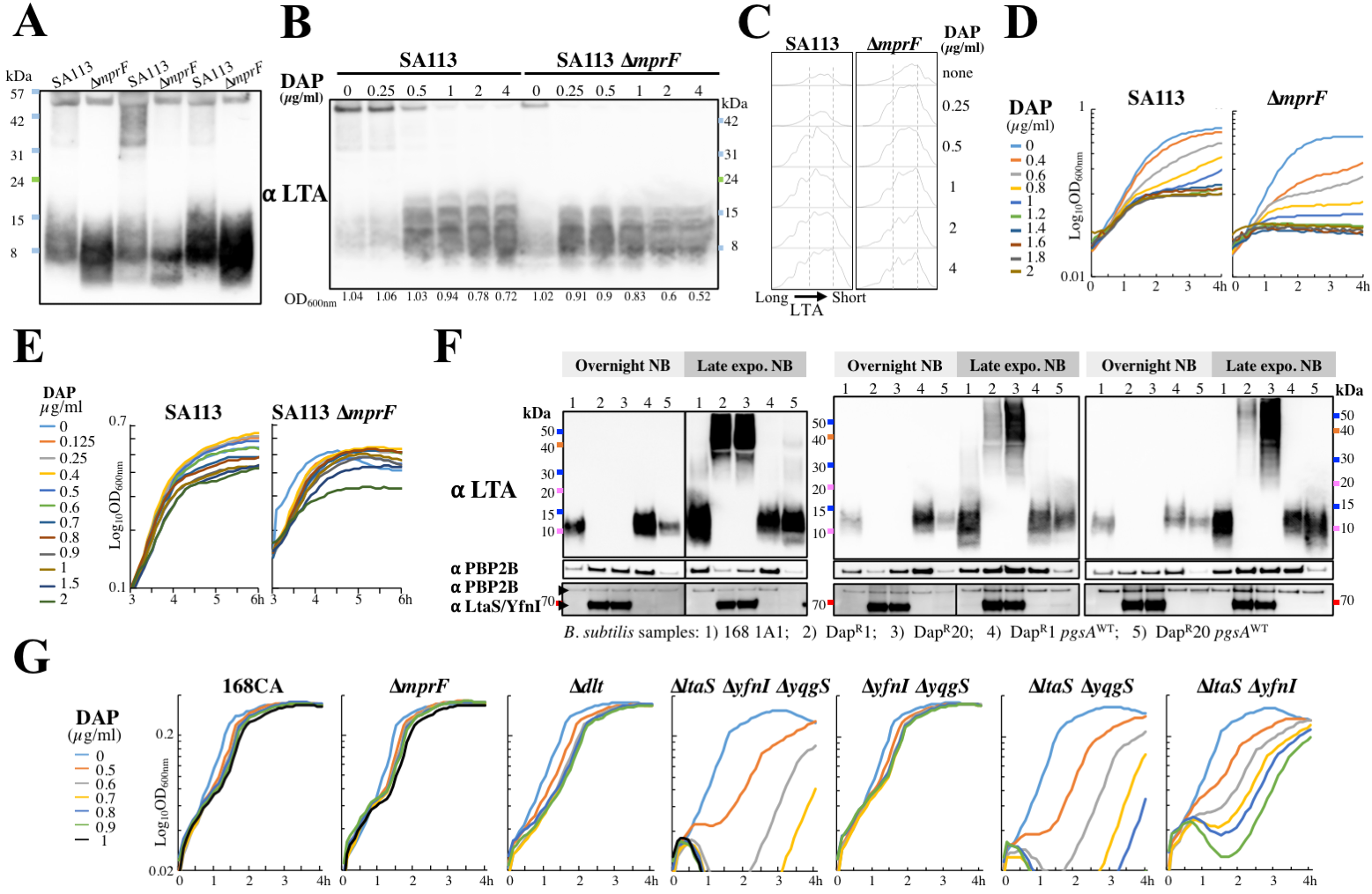

Supplement: FIG S10 [file mbio.02667-22-s0010.tif]
